# Podosome-Driven Defect Development in Lamellar Bone under the Conditions of Senile Osteoporosis Observed at the Nanometer Scale

**DOI:** 10.1021/acsbiomaterials.0c01493

**Published:** 2021-05-03

**Authors:** Paul Simon, Wolfgang Pompe, Manfred Bobeth, Hartmut Worch, Rüdiger Kniep, Petr Formanek, Anne Hild, Sabine Wenisch, Elena Sturm

**Affiliations:** † Max-Planck-Institut für Chemische Physik fester Stoffe, Nöthnitzer Str. 40, 01187 Dresden, Germany; ‡ Technical University of Dresden, Institute of Materials Science, 01069 Dresden, Germany; § Leibniz-Institut für Polymerforschung Dresden e.V., Hohe Straße 6, 01069 Dresden, Germany; ∥ Clinical Anatomy, Clinic of Small Animals, Justus-Liebig-University, 35385 Giessen, Germany; ⊥ University of Konstanz, Physical Chemistry, POB 714, D-78457 Konstanz, Germany

**Keywords:** bone, human, femur, trabecula, senile osteoporosis, TEM, SEM, ultrastructure, podosome

## Abstract

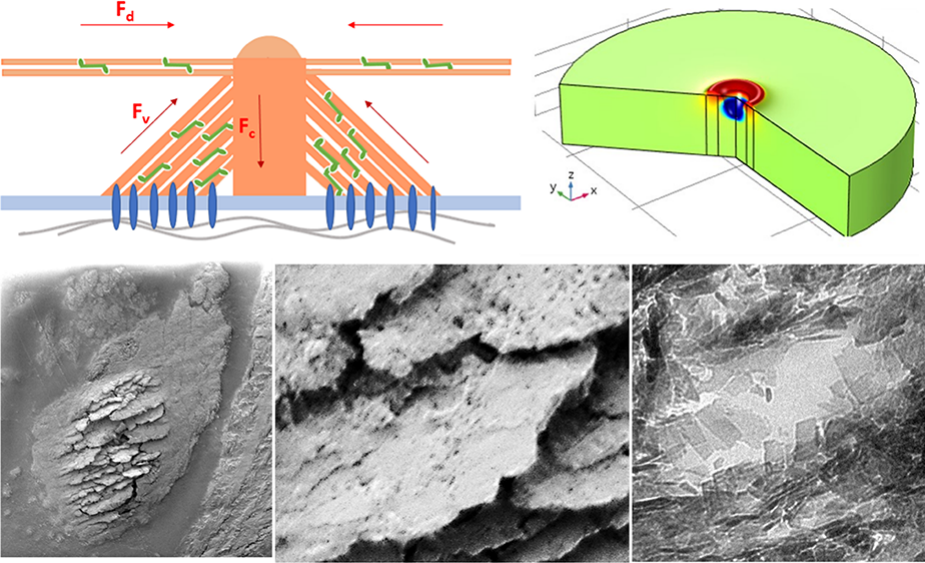

The degradation mechanism of human trabecular bone harvested from the central part of the femoral head of a patient with a fragility fracture of the femoral neck under conditions of senile osteoporosis was investigated by high-resolution electron microscopy. As evidenced by light microscopy, there is a disturbance of bone metabolism leading to severe and irreparable damages to the bone structure. These defects are evoked by osteoclasts and thus podosome activity. Podosomes create typical pit marks and holes of about 300–400 nm in diameter on the bone surface. Detailed analysis of the stress field caused by the podosomes in the extracellular bone matrix was performed. The calculations yielded maximum stress in the range of few megapascals resulting in formation of microcracks around the podosomes. Disintegration of hydroxyapatite and free lying collagen fibrils were observed at the edges of the plywood structure of the bone lamella. At the ultimate state, the disintegration of the mineralized collagen fibrils to a gelatinous matrix comes along with a delamination of the apatite nanoplatelets resulting in a brittle, porous bone structure. The nanoplatelets aggregate to big hydroxyapatite plates with a size of up to 10 x 20 μm^2^. The enhanced plate growth can be explained by the interaction of two mechanisms in the ruffled border zone: the accumulation of delaminated hydroxyapatite nanoplatelets near clusters of podosomes and the accelerated nucleation and random growth of HAP nanoplatelets due to a nonsufficient concentration of process-directing carboxylated osteocalcin cOC.

## Introduction

Numerous studies in the literature^1−8^ have been devoted to the elucidation of the biochemical and cellular processes causing the senile osteoporosis. There are only few studies devoted to the changes of osteoporotic bone on the nanoscale (1 μm–10 nm).^9−11^ Fractures of the femoral neck, vertebrae, and distal radius—being the hallmarks of osteoporosis—are the results of low energy trauma and occur almost exclusively in the geriatric population (Cummings and Melton,^12^ Riggs and Melton,^13^ Acros *et al.*,^14^ and McNamara^15^). Several ideas were developed, e.g., by the use of *in vitro* isolated or differentiated osteoclasts to recover bone or by donation of bisphosphonates to stop bone degradation.^16^ Additional healing concepts make use of the application of bone replacement materials, which can be loaded by vascular endothelial growth factors, bisphosphonates, antibiotics, and chemical attractors.^14,17,18^ In 1993, it was stated by Bailey *et al*.^1^ that collagen is modified in osteoporotic bone. Further investigations revealed that also the amount of collagen I is decreased.^1,19^ Furthermore, it is assumed that a change of the amount of collagen crosslinks, such as homocysteine, lysine, pyrrolidine, glycosylated compounds, and others, may provoke a decrease in the mechanical stability of bone.^16,20^

Recently, Yu and coworkers observed that the osteoblasts in artificially evoked osteoporotic bone of rats show distinct morphological differences to osteoblasts in healthy bone.^2^ As shown by Ozasa *et al*.,^8^ osteoporosis changes the collagen/apatite orientation and the Young’s modulus in vertebral cortical bone of rats. By various electron microscopy techniques, structural changes in osteoporotic bone could be identified with a lateral resolution down to about 5 nm for the podosome structure and atomic resolution in the case of apatite and the underlying collagen structure within osteoporotic bone. Transmission electron microscopy (TEM) on trabecular osteoporotic bone carried out by Rubin *et al.*(9) showed the occurrence of crystalline HAP nanoplatelets with irregular edges. However, there was no substantial difference in crystal length or crystal thickness between normal and osteoporotic trabecular bone. By scanning electron microscopy (SEM), the bone mineralization density (BMD) distribution and thus the mineralization process have been tracked by using the backscattered electrons with a lateral resolution of about 1 μm.^3^ In osteoporotic rat trabecula, the release of collagen and calcium degradation were observed by time of flight secondary ion mass spectrometry (TOF-SIMS) with a lateral resolution of about 1 μm.^21^ Both methods deliver an overview about the degradation extent at the microscale.

It is the intention of this study to demonstrate that electron microscopy can offer useful additional information to the defect structure of osteoporotic trabecular bone, particularly focused on the formation of microcracks and their aggregation to larger defects. In the following, we use high-resolution TEM and Fourier transform analysis of the high-resolution images combined with energy-filtered TEM for elemental mapping for a detailed analysis of nanosized bone defects in lamellar bone in the case of senile osteoporosis and discuss possible defect formation mechanisms.

## Results

**Figure 1:**
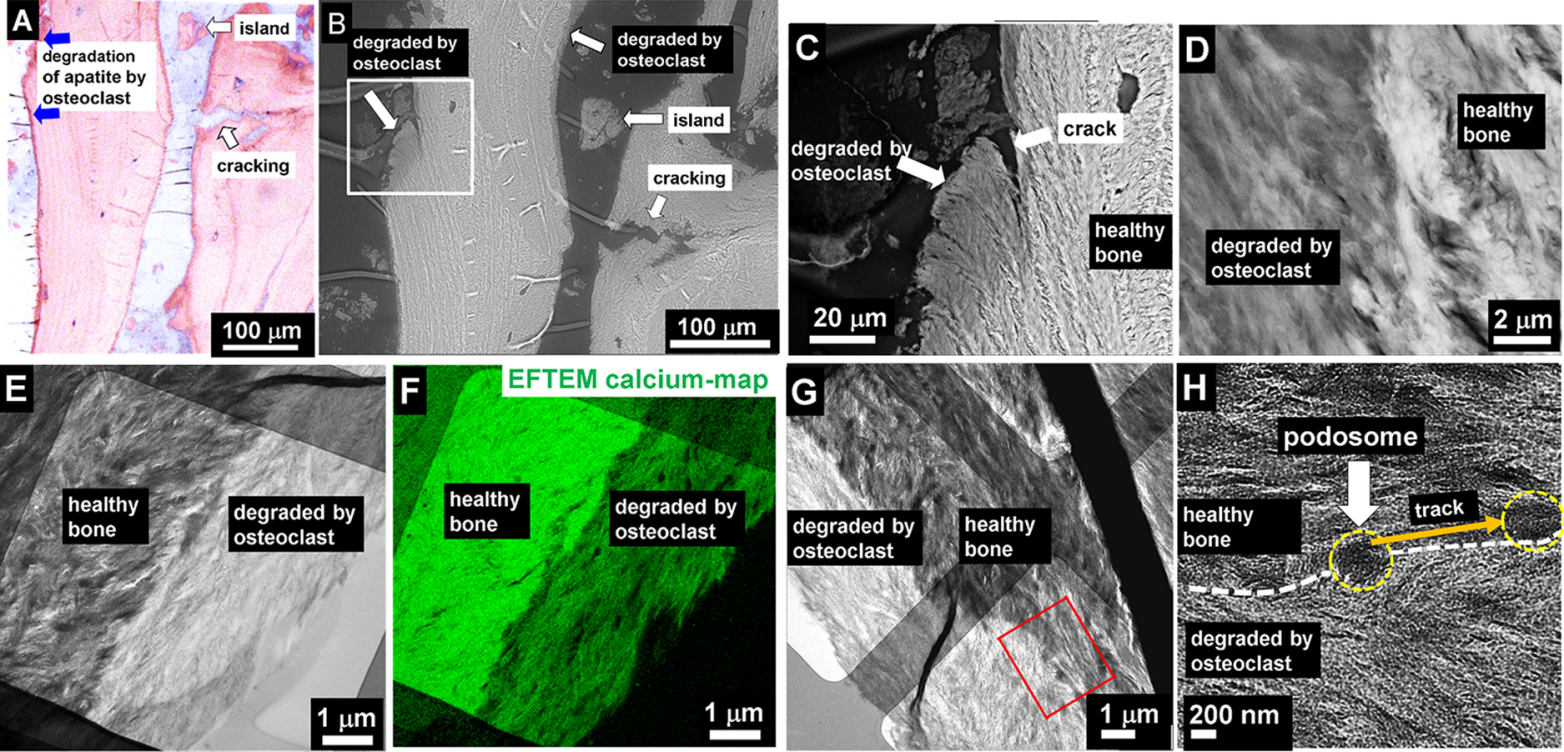
(A) Light microscopy image of fractured trabecular bone of a 67 years male after staining, according to Trump^23^ and Ito.^24^ The lamellar bone is surrounded on the left side by a small zone of osteoid (dark pink, blue arrows). Intensively stained lines within the bone samples refer to osseous transformations in the course of remodeling. Osteoblasts, bone lining cells, and osteoclasts are not present, while cavities of about 10 μm indicate the positions of osteocytes. (B) SEM image of the same region shown in (A) with etched patterns caused by osteoclast activity. The patterns appear as dark areas at the edges. (C) SEM image of a trabecula edge. The dark region indicates less calcium content, corresponding to dissolution of the inorganic component HAP. This weakening leads to cracks. (D) SEM at a higher magnification reveals the thinner edge, appearing dark at the left. (E) TEM of the edge shows reverse contrast. The thin edge at the right is etched and looks brighter than the unaffected bone material at the left. (F) The calcium map shows the high calcium density of bone at the left side, whereas the vicinal area at the right side shows the large material lost. (G) Overview TEM image of dissolved edge. (H) Enlarged image taken from (G) marked by a red rectangle at bottom right. Due to the magnification and thus lens current change in TEM, an image rotation of about 45 degrees has to be considered between images (G) and (H). At the interface (white dashed line) between the bone and etched region, there is no remarkable difference in collagen fibril structure observed. Also, the collagen striation period of 67 nm is preserved. Interaction traces (orange arrow) of a single podosome are seen at the center (marked by yellow circles).

In contrast to healthy bone of young individuals, the trabecular bone of older people (Figure 1A and Suppl. Fig. 1) is nearly devoid of osteoid. Also, cell activities are much less than in inflammation zones of fractured bones in younger individuals. In Figure 1A,B the sublayer structure of the plywood bone lamella with a periodicity of about 5 μm is visible.^22^ Histologically, (staining according to Trump^23^ and Ito^24^) the red colored areas of trabecular bone correspond to lamellar bone, appearing bright pink, which is sometimes surrounded by a small edge of osteoid (dark pink). In contrast, e.g*.*, to inflammation zones of fractured bone of young individuals, no remarkable cell activity (neither osteoblasts nor osteoclasts) can be detected. The images in Figure 1B–H reveal that bone starts to be damaged due to stepwise resorption and migration of osteoclasts at the edges and along surfaces. The dark band at the edge of about 5 μm in width contains less calcium phosphate than the bright inner main part of the piece of unaffected healthy bone. In the migration zone, a trace of a thin layer of a gradually degraded extracellular matrix (ECM) can be observed (for more details, see Discussion, “Degradation Caused by Individual Podosomes”).

Figure 1C shows an SEM image at a higher magnification of this region marked by the white rectangle in Figure 1B. The healthy bone on the right is bright, different to the damaged thinner area on the left, which is darker. At the upper part of the edge a thin dark region of 1–3 μm in diameter is observed, indicating a dissolution process. The large 20–30 μm sized dark area at the bottom with detached and irregularly shaped remnants of calcium phosphate at its top is more heavily affected. The lower part is already separated from the massive piece of bone (see arrows). The enlarged micrograph of the interface in Figure 1D (marked with the right arrow in Figure 1C) demonstrates the mass loss at the left-hand side of the edge area compared to the healthy part on the right. The TEM bright-field micrograph of the edge reveals a degradation zone running parallel to the edge with about 3 μm in width (Figure 1E). TEM investigations (Figure 1E–G) carried out on the same area as for SEM (Figure 1C,D) show the same phenomenon, however with inverted contrast in comparison to SEM in backscattered mode. In SEM, a higher amount of calcium phosphate is indicated by a higher brightness, whereas in TEM, a higher mass density is denoted by the dark appearance due to larger scattering. At the same area, elemental calcium mapping by energy-filtered TEM (EFTEM) was performed, where the presence of calcium is indicated by green (Figure 1F). A thin layer of HAP together with collagen is removed, leading to a clear vertical thickness step in the center of the image of about 160 nm, assuming a thickness of the healthy bone of about 220 and 80 nm for the degraded part, estimated from the gray scale line profile. Regardless of the massive degradation at the edge, the bone structure is not altered. As revealed by TEM at a higher magnification, the fibril structure and the striation pattern are preserved (Figure 1G,H). Figure 1H is the zoomed area marked by a red frame in Figure 1G, representing a zoom into the interface of healthy and damaged bone marked by a white dashed line. Since there is a magnification change between Figure 1G and Figure 1H, an image rotation occurs due to lens current changes. Thus, a rotation angle of about 45 degrees between the images has to be considered. In the center of Figure 1H, an “interaction zone” of a single podosome can be seen (marked by an arrow and a yellow dashed circle). This shallow zone is caused by the interaction of a podosome with the extracellular bone matrix. The ECM-degrading podosomes in resorbing osteoclasts are also referred to as invasive podosomes.^25^

These small protrusive F-actin based structures are distributed at the ventral cell membrane in the so-called ruffle border and are extended into the underlying ECM. The shallow pit is formed by the lytic activity of the central actin core of the invasive podosome (diameter of about 300–400 nm).^26,27^ It is partially covered with actin filament residues of the actin ring (thickness of 200–300 nm) and the central core (diameter of about 150–200 nm) of the podosome^28^ (see also Figure 6A; for more details, see Discussion and Supporting Information). Podosomes are dynamic structures of the osteoclast, which fulfill essential functions in the cell adhesion, mechanosensing, and degradation of the bone matrix.^25,28−31^ Individual podosomes possess a lifespan in the range of 2–10 min.^28^

At the inner side of the ventral membrane the ruffled border is closely surrounded by the sealing zone. In the sealing zone, also closely packed podosomes are situated. The sealing zone is also connected with the basolateral side of the cell via the cytoskeleton.^25^

### Degradation Structure Caused by Individual Podosomes

**Figure 2:**
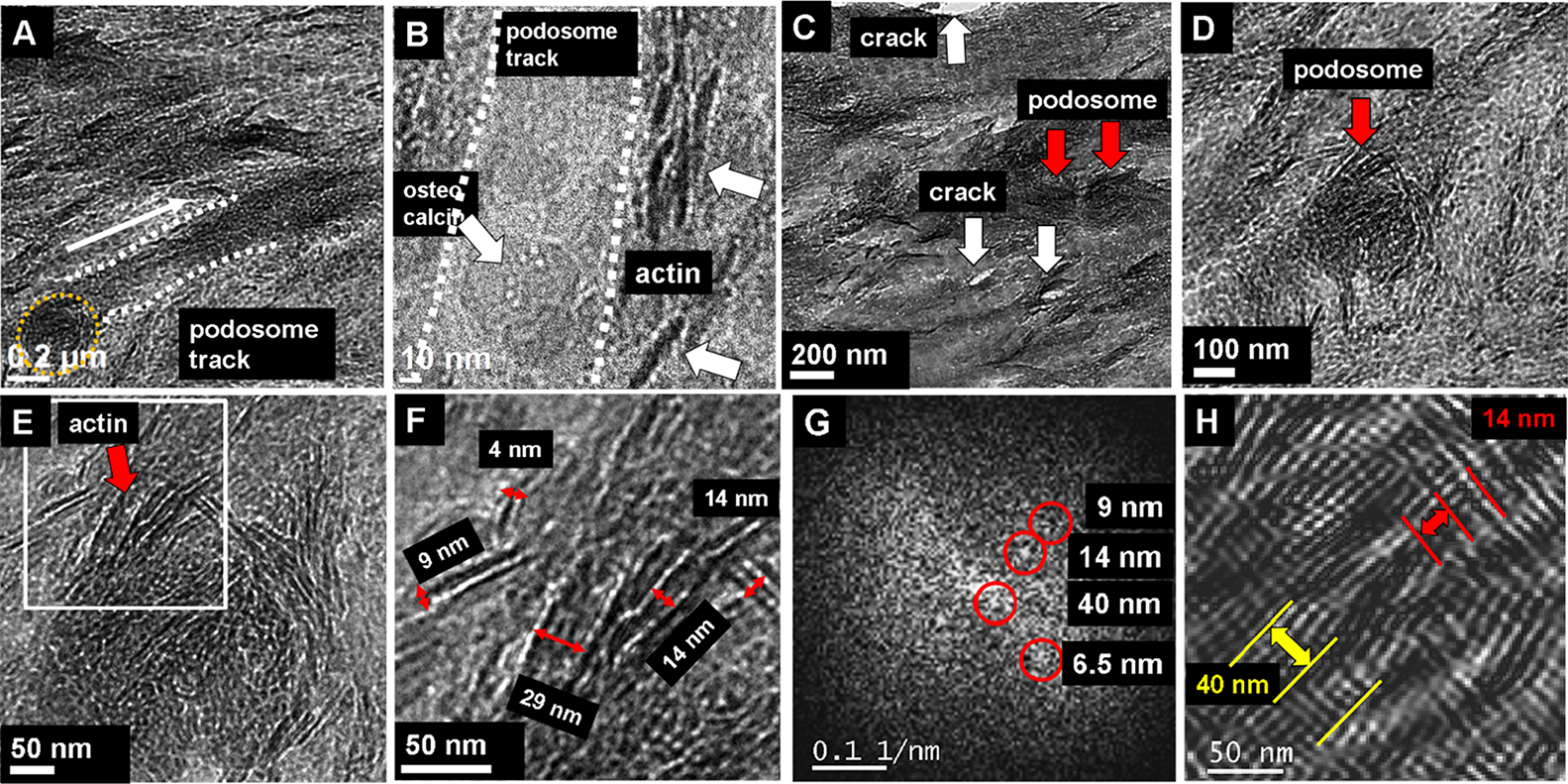
Podosome migration. (A) Track of podosome (yellow circle) movement, see white arrow. (B) Zoom into the podosome track (see white dotted lines) where the surface material of the lamella is removed. Right and left remnant actin filaments are observed. (C) Trace of a single podosome (marked by red arrows) on the mineralized extracellular matrix. The shallow pit caused by the lytic activity of the central actin core of the podosome pit is covered with the filament residues of the actin core and the actin ring surrounding the central core. At a larger distance, mineralized collagen microfibrils are visible. Along the fibrils, microcracks (white arrows) as well as HAP plates were found. (D) Overview of a single podosome pit (red arrow) with a diameter of about 340 nm, appearing as dark disc in the center of the micrograph. (E, F) Further zoom into the podosome pit reveals actin filaments of about 4–9 nm in diameter in the center and 9–14 nm at the periphery. (G) Fast Fourier transform of (F) showing reflections of periodic ordering of fibrils. (H) Fourier-filtered image of the central part of the podosome pit in (E). The 40 nm lattice represents loose actin filaments with α-actinin spacer (contractile bundle), whereas the 14–16 nm periodicity indicates tight packing containing filamin and fimbrin. The loose and tight packed fibrils are normal to each other and form a nanopattern. The dots at the top center are individual globular actin monomers with about 4 nm in diameter.

Figure 2A shows the interaction of an invasive podosome with the ECM. The osteoclast migrates along a trace running parallel to a mineralized collagen microfibril. The trace is cleaned by partial ECM degradation and gives free sight on singular hydroxyapatite platelets along the collagen fibril (Figure 2B). Outside the track, remnants of actin filaments are observed (arrows on the right). For zoom series, see Suppl. Fig. 2. Figure 2C shows an overview of podosome pits (dark discs, see, e.g., red arrows).

Due to the lytic activity in the resorption lacuna (see also few details in Discussion, “Degradation Caused by Individual Podosomes”) together with the local stress field of the podosome, the bone structure is weakened. The induced force results in microcrack formation visible as bright rod-like structures marked with white arrows in Figure 2C emerging around the podosome site. They are grown along interfaces of HAP platelets oriented perpendicularly to the image plane (short dark strips in the TEM image). The zoom into a podosome reveals the presence of G-actin and actin filaments of different diameters (Figure 2D–F). The Fourier transform (Figure 2G) and the corresponding filtered image (Figure 2H) suggest diameters of globular actin of about 5–6 nm, filamentous actin of about 7 nm, fimbrin-linked and α-actinin-linked actin filaments of about 14 and about 40 nm, respectively. More details of the used filtering procedure are given by Simon *et al.*.^32^

**Figure 3:**
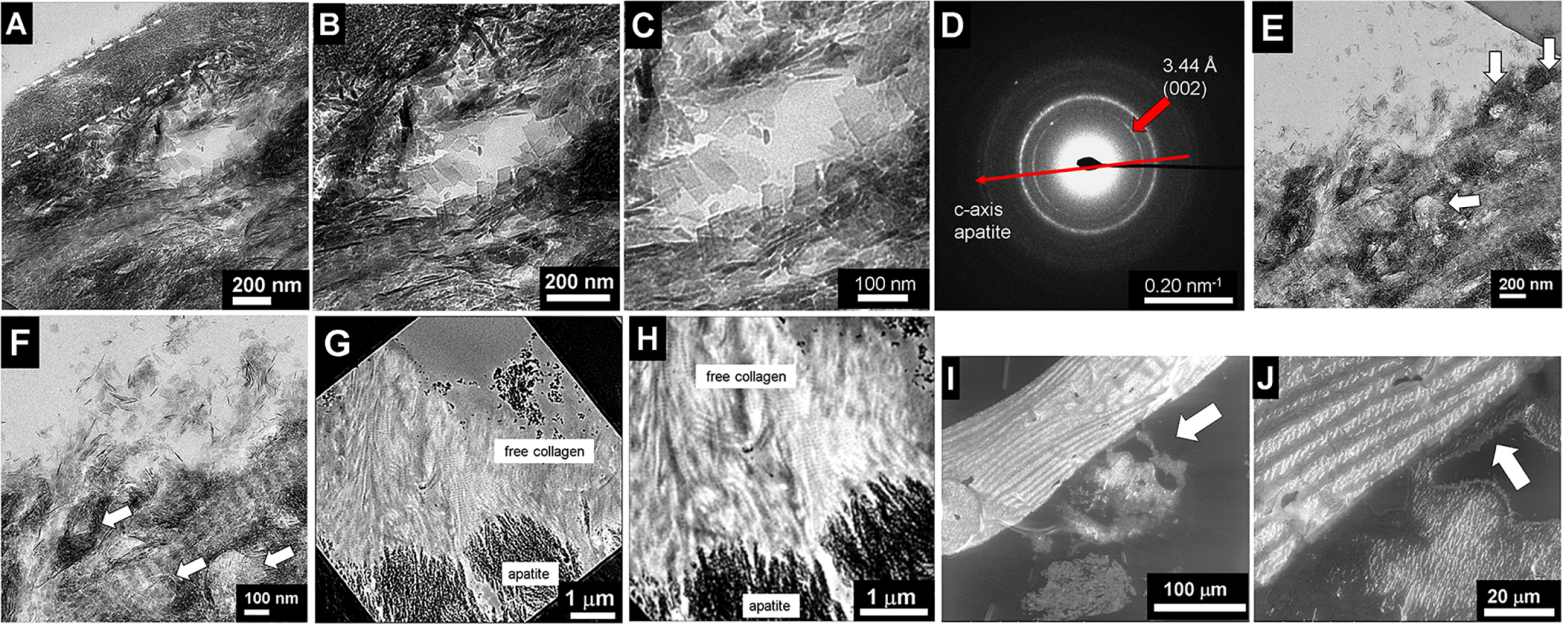
TEM and SEM imaging of local destruction in a massive piece of the trabecula due to podosome activity. (A) Invasive podosomes generate holes in the bone piece. Here, a single pit is shown with a diameter of about 300 nm, appearing as a bright area in the center of the micrograph. The edge of the trabecula just above the hole is also damaged (white dashed lines). (B) Enlargement reveals free standing HAP platelets inside the disruption zone around the hole, indicating the loss of the supporting collagen matrix. The plates are larger than expected, the largest are over 100 nm long and show 60–80 nm in width. The collagen fibrils underneath, showing typical striation pattern, are still present and are not affected by the local removal of collagen in the neighbored area. (C) Zoom in to the plates. (D) Electron diffraction shows the presence of HAP. (E) Decomposition of the trabecula observed at the edge of the bone sample. Massive presence of pits generated by podosomes (some marked by arrows). (F) The zoom shows that the HAP depletion takes place parallel to the collagen fibril long axis. At the top, apatite platelets are set free due to collagen degradation. (G, H) Degradation due to partial dissolution of the apatite along the rim yields free lying collagen fibrils. (I) Scanning electron microscopy (SEM) image (secondary electrons) of a trabecula reveals layer-by-layer depletion of the plywood structure. Depletion of organics becomes evident, appearing as a bright irregular assembly below the trabecula (see arrow). (J) The thin layer (marked with an arrow) is partially detached from the trabecula surface and shows less HAP content.

The local dissolution of the ECM in the trabecula occurred as detected by TEM (Figure 3A), leading to the generation of holes and pores, partially filled with remnant loose HAP platelets at the rim. The size of the pore is about 200 nm, thus slightly less than the podosome diameter. The edge of the bone piece (marked by white dashed lines in Figure 3A) in the vicinity of the pore is thinned in such a way that the HAP plates and the collagen striation are not present anymore. At this thin edge, only a fine network of thin fibrils with few nanometer diameters remains. Remarkably, while the supporting collagen matrix for the HAP platelets is destroyed, any HAP platelets are partially free standing fixed by neighboring plates (Figure 3B,C). They are considerably larger than known plate sizes in healthy bone of about 45 x 20 nm. In our case, the largest plates are about 100–120 nm in length and 60–80 nm in width. The HAP orientation along the fibrils still persists and is still parallel as proved by electron diffraction (Figure 3D). Figure 3E,F shows that degradation of the ECM takes place parallel to collagen fibril long axis caused by high activity of invasive podosomes, see arrows. The bright structures are the imprints of the ECM degradation caused by the posodomes. The ECM degradation due to podosomes along the edges leads to a thinner apatite rim. This eventually results in the uncovering of the collagen scaffold, see Figure 3G,H. At the bottom of the images, remnants of apatite (dark regions) are found, whereas above, a free lying and dense collagen fibril matrix appears. A layer-by-layer depletion of the plywood structure is revealed by scanning electron microscopy (SEM) on a piece of trabecular bone in Figure 3I,J. Due to the surface sensitivity of the secondary electrons, the depletion of collagen becomes evident, appearing as irregularly shaped bright regions just below the trabecula, see arrow in Figure 3I. Only the part directly attached to the bone piece is layered, whereas the detached part is denatured and does not possess the fibrillar structure any more. In the enlarged view of the depletion region at the top right, the lower layer (Figure 3J, arrow) is partially detached from the trabecula surface and shows only less HAP content.

### Degradation Structure Caused by Podosome Assemblies

**Figure 4:**
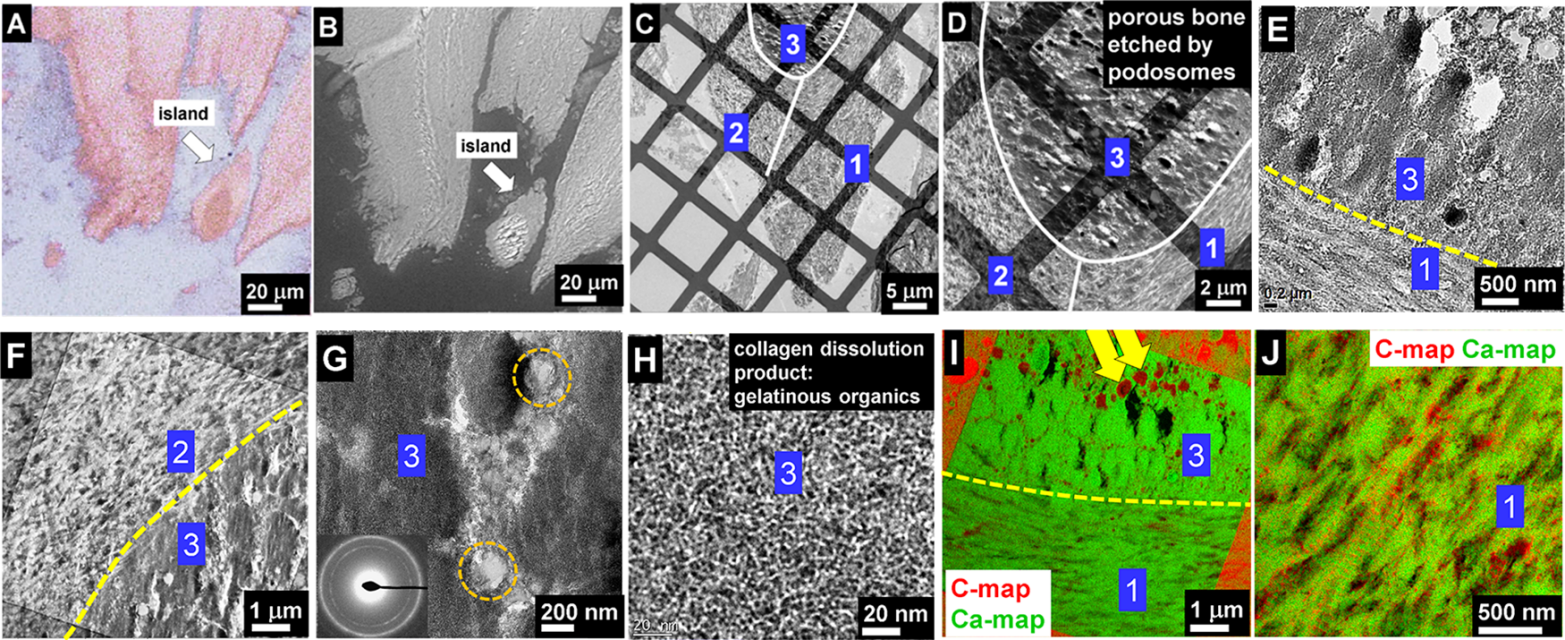
Light microscopy (LM), scanning electron microscopy (SEM) and TEM micrograph display three regions of different decomposition of the “island”. (A) Light microscopy of stained sample shows the island (arrow) in the vicinity of massive pieces of bone. (B) SEM of the same region. (C) TEM overview image of the island with three different heavy degradation zones. TEM image is rotated by 180 degrees and mirrored with respect to LM and SEM images in (A) and (B). (D) In region 3, the collagen fibrils are completely dissolved. A gelatinous collagen network is formed. In region 2, the collagen fibrils are heavily affected. Region 1 is the most preserved structure with nicely ordered fibrils, already with incomplete coverage of HAP platelets. (E–H) TEM of region 3. (E) Overview image of the interface between region 3 on the left with holes and region 1. (F) Enlarged interface of region 2 with heavily affected fibrils and region 3 where the fibril structure is destroyed. (G) High resolved image of region 3 showing pores and podosome pits with 800 nm distance (orange circles). The track between them is a shallow trace of ECM dissolution and thus paved with cracks. (H) Degenerated collagen remnant due to enzymatic and lytic podosome activity: image of the gelatinous organic layer in region 3, composed of a disordered network of fibrils with diameters of 1–2 nm. (I) Energy-filtered TEM of regions 3 and 1. Region 3 shows significant lack of collagen fibrils and loss of material in the form of pores (black areas at the top). Yellow arrows indicate the presence of organics, presumably actin, in the smaller pores. Region 1 reveals the most preserved structure. (J) The combined carbon (red) and calcium (green) map of region 1 at high resolution reveals that, in this region, mainly HAP is present. The collagen content is strongly reduced, mainly present in the center. The Ca distribution is inhomogeneous due to acidic depletion (dark areas).

As shown in Figure 4, besides degradation by individual podosomes, there are interaction zones of podosome clusters with the extracellular matrix. Following a classification proposed by Linder and Wiesner,^28^ we can distinguish podosome clusters, rings, and the densely packed sealing zone (SZ), which confines the resorption lacuna and/or SZ-like belts (SZL). Typical distances of the individual podosomes in clusters are about 750 nm, in the SZ about 210 nm. The clusters are stable patterns (up to several hours), while the lifespan of an individual podosome within a cluster is around 3 min in average and varies in the range of 2–10 min. The cluster size can reach up to 3 μm. The rings are transient forms in the transition from clusters to the stable SZ. In the ring structures, the lifespan of individual podosomes is shortened from 3 to 1 min. Thus, it can be concluded that the observed larger degradation patterns shown in Figure 4 are produced by a podosome cluster.

In the stained light microscopy micrograph in Figure 4A, an “island” is shown and in Figure 4B the corresponding SEM image. In the TEM images of the “island” (Figure 4C,D), three different parts are visible. On the left, named as region 2, a degraded structure is observed. The collagen fibrils are already partially decomposed to such an extent that they do not show the striation of 67 nm. In the center of the island (region 3), the heaviest destruction appeared. The organic part is affected in such a way that the collagen matrix function is lost. The apatite platelets start to fall apart as demonstrated by broken pieces protruding from the surface. The apatite platelets do not show a regular tetragonal habit, and they are rounded and irregularly shaped. The apatite is disordered as shown by electron diffraction, and no collagen fibrils are present. Region 1 represents the most preserved structure. The collagen fibrils are clearly imaged with the striation; however, the coverage by HAP is not complete any more. The island was originally a part of massive bone. Due to localized formation of a cluster or a band of invasive podosomes inside a ring-like sealing zone, such extreme damage can be explained (see also the discussion at pages 21–23). Figure 4E shows a higher ring-like magnification of the interface between region 3 on the left and region 1 on the right. The interface between regions 2 and 3 is very sharp (Figure 4F) due to the distinct structural changes between the two regions. Further zoom into the heavily damaged HAP plates of region 3 reveals pits and holes evoked by podosomes (Figure 4G). Two pits with 800 nm distance are highlighted by orange circles. The track between them is thinned out, and cracks evolve along the migration path. The apatite component shows a random orientation as indicated by the electron diffraction inset. As shown in Figure 4H, in region 3, the collagen component is heavily destroyed and disintegrated. A mesh of very fine fibrils down to the thickness of a triple helix (1.5 nm) and alpha chains (0.8 nm) covers the surface of this area, giving rise to a gelatinous organic matrix.

In order to prove that the osteoclast activity causes HAP and collagen dissolution, energy-filtered transmission electron microscopy (EFTEM) was performed on regions 3 and 1 (Figure 4I,J). The calcium and carbon absorbance edges were used to record the elemental distribution. The overlaid calcium and carbon maps in Figure 4I of regions 3 and 1 show that mainly HAP is present (green), whereas collagen is depleted (red). Organics is mainly present in region 3, see bright red spots at the top and remnants appearing as fine and faint red lines between roundish HAP plates. The calcium map indicates a remarkable loss of HAP and thus bone mass in region 3, evidenced by black holes and dark lines between HAP plates. In region 1, a large number of fibrils appear black, indicating a demineralization process by the removal of HAP. As we have already shown for region 3, the collagen structure is destroyed (Figure 4H), whereas region 1 still shows preserved collagen fibrils. In the combined carbon-calcium map, at a higher resolution of region 1 (Figure 4J), mainly HAP (green) is present, with some residues of collagen in the center (red). HAP is strongly affected by acidic etching (dark areas). At thinner areas, collagen striation of 67 nm is visible through the calcium signal. Massive HAP plates of about 200–300 nm in size are observed at the top. The total amount of collagen is remarkably reduced, and only some partially free lying fibrils in the center (red) (Figure 4J) remain. HAP is observed over the whole area. However, the calcium distribution is inhomogeneous due to a structured degradation process caused by a heterogeneous distribution of solitary podosomes in clusters at the ruffled border of the resorption lacuna. For a complete series of elemental maps and corresponding bright-field images, see Suppl. Fig. 3.

**Figure 5:**
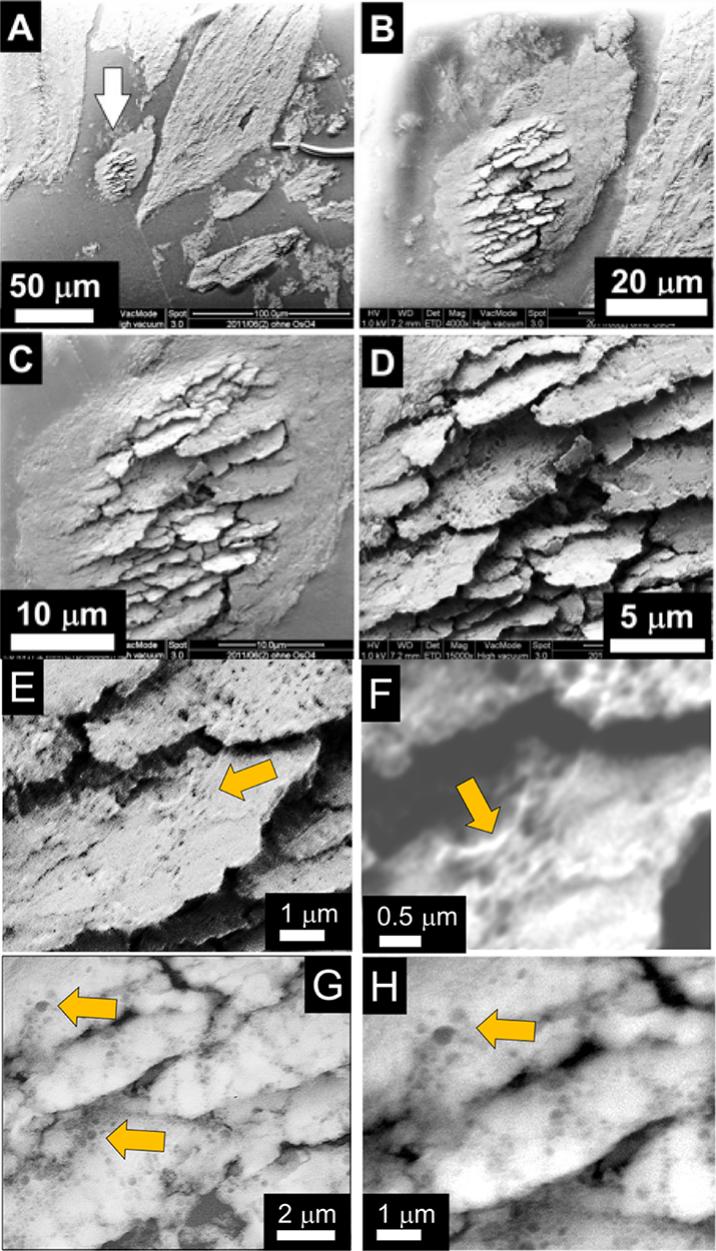
SEM zoom series of an isolated and heavily affected “island” shaped piece of bone recorded with surface-sensitive secondary electrons. (A) SEM overview image with the island marked by an arrow. Large intact fragments are detached from the trabecula, but also irregularly shaped pieces are observed. (B) On the right, a healthy piece of bone displays a plywood pattern, which does not show up any more in the isolated piece. (C) Enlargement reveals the central region where brittle HAP platelets protrude from the surface, whereas at the edges, the sample seems to be not affected. (D) The highest magnification used reveals that the zone is free of any collagen fibrils, which results in a very fragile structure. (E, F) Topography-sensitive secondary images display the presence of actin (see arrow) and thus indicate the activity of invasive podosomes on the surface of the HAP plates, causing the observed nanocavities. The HAP dissolution is evoked by the high acidity (H^+^ ions) in the resorption lacuna. The diameter of the nanocavities is in the range of the podosome diameter (see Figure 2D) and corresponds to pit sizes observed by TEM (Figure 3A–D). (G, H) Material-sensitive backscattered images reveal a mesh-like distribution of nanosized cavities on the surface with diameters of 100–400 nm; mesh width about 600–800 nm.

Figure 5A shows SEM images of distinct structures, which stem from the isolated “island” (marked by the arrow), see also Figure 4 from the same region. This part displays an advanced stage of degradation and is separated from the massive bone on the right showing the regular plywood pattern. The zoomed micrographs in Figure 5B–D show large HAP plates, which protrude from the surface in the central part of the “island”. At an even higher magnification (Figure 5E,F), it seems that the big plates are grown by aggregation and sintering of randomly oriented small HAP platelets. On the surface of individual plates, traces of actin fibrils (see arrow) and cavities were observed. The organics is spread like a spider net on the HAP plates, forming an incomplete coverage (Figure 5F, arrow). In order to characterize the nanocavities, the backscattered mode was chosen, revealing pits of 100–400 nm in diameter (Figure 5G,H). These diameter values are in the range of the pit diameters of individual podosomes registered also by TEM (Figure 2C,D, Figure 3A–F). Often, the pits are arranged in short chain-like traces with a distance between neighboring pits of about 500–800 nm (Figure 5E–H). Interestingly, the nanocavities only appear on the brittle HAP plates on which also actin is observed. Free lying HAP areas without covering by actin are not affected.

## Discussion

### Degradation Patterns on the Micrometer Scale

In Figures 1–5, characteristic features of degradation patterns on the microstructural level are shown. As stated by Ozasa *et al*., changes in the micro-orientations of collagen and apatite deteriorate bone strength in osteoporosis.^8^ There are also more pronounced defects in the plywood structure of the lamellae stacks in osteoporotic bone as shown by Rubin and Jasiuk in 2005^33^ (see, for instance, Figs. 3 and 7 in ref (33)). These defects are also responsible for the differences in the microstructural degradation patterns at the bone surface. For instance, there are surface regions covered with more or less ordered mineralized collagen fibrils (Figures 1, 2, and 5), alternating with regions with a completely destroyed fibril structure (Figure 3), and with “islands” of packages of HAP (Figure 5).

### Structural Changes on the Nanometer Scale

Characteristic structural features of the interaction of osteoclasts with the mineralized extracellular matrix (ECM) are summarized in Figure 2A,B and Suppl. Fig. 2. They show the transition from a pit to a continuous trace. As known for healthy trabecular bone (see, for instance, Gentzsch *et al.*(34)), the resorption lacunae are arranged parallel to collagen fibers, which correlates with the dominating direction of the tensile strain.

For the interpretation of the observed structural changes in a resorption lacuna due to cellular activity on the nanoscale, a more detailed analysis of the traces caused by invasive podosomes is helpful.

The cycling motion of invasive podosomes is directly linked with the degradation of the extracellular matrix. Invasive podosomes degrade the ECM by binding matrix-lytic enzymes, particularly by metalloproteinases such as matrix metalloproteinases (MMPs) or ADAMs (a disintegrin and metalloproteinase).^26−28^ Furthermore, they transfer the force generated by actin assembly to form protrusions and cross tissue barriers.^35^ All these data demonstrate that podosomes transmit endogenous forces to the outside ECM proteins.^36^ Looking on the damage zones with a higher resolution, as in Figures 1H,2A–H, 4E–H, and 5E–H, and Suppl. Fig. 2, various patterns of discrete damage zones are visible. These pits with diameters typically from 100 to 400 nm have been identified as traces of the interaction of individual podosomes with the underlying bone matrix (Linder *et al.*,^28^ Akisaka *et al.*,^37^ Takito *et al.*,^31^ Collin *et al.*,^36^ and Schachtner *et al.*(35)).

### Degradation Caused by Individual Podosomes

**Figure 6:**
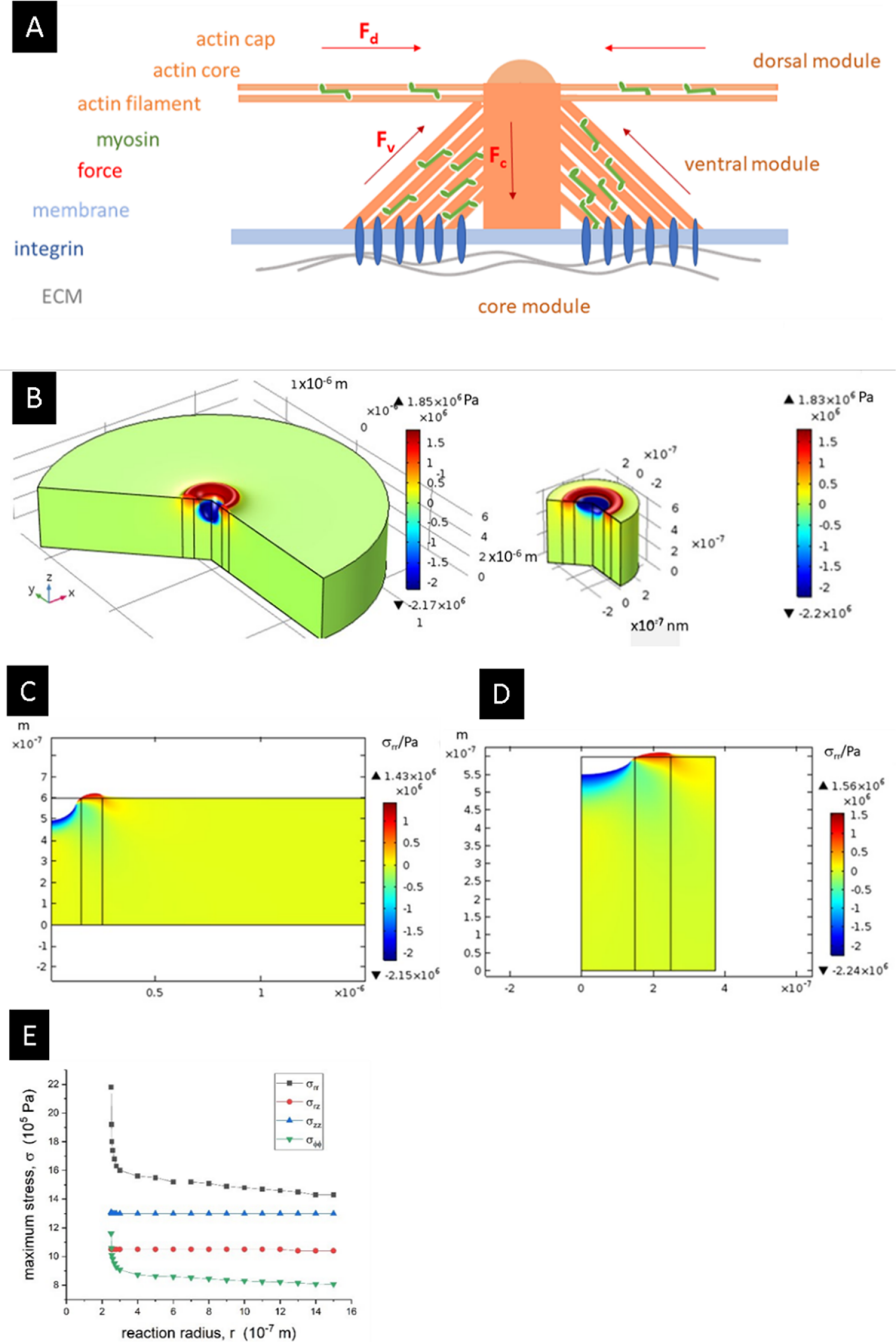
Stress distribution in the extracellular matrix near the surface caused by podosomes. Stresses were calculated within an axial podosome model assuming isotropic elastic properties of the lamellar bone with plywood structure. (A) Structural model of a podosome, typically 0.5 μm in diameter. (B) Presentation of the first principal stress σ_prin_ (*r*,*z*) of a solitary podosome, reaction range typically 3 μm in diameter (left), and a podosome in a cluster with a podosome reaction range of 750 nm (right). The force *F*_c_ = 150 nN acts within the central core (diameter of 0.3 μm) on the extracellular matrix. (C) Distribution of the radial stress σ_rr_ (*r*,*z*), podosome reaction range 3 μm. (D) Distribution of the radial stress σ_rr_ (*r*,*z*), podosome reaction range 750 nm. (E) Maximum values of the various stress components in dependence on the podosome reaction range 2 r. Maximum values of the various stress components in dependence on the podosome reaction range 2 r, assuming cylindrical symmetry along the podosome axis (σ_rr_ = radial stress, σ_rz_ = shear stress, σ_zz_ = axial stress, σ_ΦΦ_ = tangential stress).

Typically, a single podosome has a diameter of 0.5–1 μm and a height of about 0.6 μm. The average distance of solitary podosomes in a cell is about 3 μm. In Figure 6A, a schema of the axial architecture of a podosome is shown as an (*r*, *z*)-cross section. To explain the various functions of a podosome, we distinguish three main components:

(i) the central protrusion module (the central part of the core) of branched actin filaments and the linear filaments forming the peripheral protrusion module (the peripheral part of the core),^38^
(ii) the ventral module consisting of interpodosomal actin filaments. It connects the core of the podosome with the plasma membrane of the osteoclast, and
(iii) the dorsal module forming a long-range connecting network with the surrounding podosomes. It is, for instance, relevant for pattern formation of podosomes as clusters, rings, and the sealing zone.^39^

In the ventral and dorsal modules, the actin filaments are crosslinked by myosin IIA motors. Each module contains specific actin interactors (e.g., actin binding as WASP, arp2/3, cortactin located at the central part of the podosome core, and α-actinin partially colocalized with vinculin at the ventral part of podosomes^38^). Podosomes are dynamic structures characterized by a cycling expansion and contraction of the podosome core in their interaction with the extracellular substrate. The motion of the actin core is caused by the superposition of the force *F*_c_ due to polymerization and dissolution of globular actin monomers at the actin filaments in the actin core with the force of the myosin motors located in the actin cables of the ventral module (force *F*_v_). The force *F*_d_ within the actin cables in the dorsal module is also caused by myosin motors. The cycling motion of invasive podosomes is directly linked with the degradation of the extracellular matrix.

As pointed out by Takito *et al.*,^31^ the distribution of invasive podosomes in the ruffled border membrane can be observed as pit-like traces on the migration path when the osteoclast has stopped bone resorption and migrates on ECM surfaces. Such a pattern is formed when the sealing zone has a ring-like shape. As seen in Figures 1C and 2A,B and Suppl. Fig. 2, also a transition from a pit-like lacuna to a continuous degradation trace of the podosome structure was observed. In this case, a crescent shape of the sealing zones can be expected. This leads to a continuous trail- or trench-type resorption trace.^31^ Osteoclasts with a crescent-shaped sealing zone form deeper resorption traces than those with a ring-shaped sealing zone.

As pointed out by Takito *et al.*,^31^ the distribution of the podosomes in the ruffled border membrane can be observed depending on the depth the resorption lacuna. This depth influence occurs, when the osteoclast changes from the stationary resorption into the migration mode. If the resorption lacuna is shallow, the osteoclast migrates without resorption along the surface until the following stationary resorption state starts. However, in the case of a deep resorption lacuna, the osteoclast cannot leave the resorption lacuna. Therefore, the wall of the lacuna is moving together with the migrating osteoclast along the surface. In conclusion, we can observe in the case of a shallow resorption lacuna the discrete pattern of the resorption pits of invasive podosomes distributed in the resorption lacuna, whereas with a deep lacuna, the osteoclast produces a trail- or trench-type trace without a sharp pattern of single podosomes. In human bone, both types of resorption traces have been observed. The pit-like trace is formed when the sealing zone has a ring-like shape.^31^ Alternatively, also a crescent shape of the sealing zones is possible. This leads to the continuous trail- or trench-type resorption traces. Osteoclasts with a crescent-shaped sealing zone show higher collagenolytic activity and form deeper resorption traces than those with a ring-shaped sealing zone.^40^ The chains of discrete resorption pits in Figures 1H, 4E, and 5E–H, allows the conclusion that, in this bone structure, ring-like sealing zones have been formed in the osteoclasts. As seen in Figures 1C and 2A,B and Suppl. Fig. 2, also a transition from a pit-like lacuna to a continuous degradation trace of the podosome structure was observed. The mode of the sealing zone depends on the cell type and the specific substrate structure.^40^ The pits as well as the continuous degradation trace are covered with actin fragments (Figure 2A–F and Suppl. Fig. 2.).

As shown in Figure 2C, few microcracks are formed near the pit. These microcracks are caused by a localized radial tension during the pit formation. In Figure 6B,C, theoretical estimates of the first principal stress σ_prin_ and the radial stress σ_rr_ at the surface of a bone lamella are plotted for the case of an individual podosome as well as for a podosome located in a podosome cluster. For the modeling, experimental data from protrusion force microscopy (PFM)^41^ studies have been used. The change of the deformation of formvar sheets caused by oscillating podosomes of living cells has been measured by atomic force microscopy. Underneath the central core of single oscillating podosomes, indentation forces up to 150 nN have been measured. These forces are caused by the combined action of myosin motors and actin polymerization in the podosome.

Assuming a maximum indentation force of 150 nN underneath the central core^41^ of the podosome, the induced stresses are in the range of hundreds of kPa up to few MPa near the podosome (for details, see the Supporting Information). Obviously, the formation of submicrometer cracks near podosomes is caused mainly by the radial tensile stress σ_rr_. The stress acts in a shallow zone beneath the surface up to a depth of about 0.1 μm. The calculated stress values are only a crude upper estimate because of our strongly simplifying assumptions concerning the used materials equations, neglecting, for example, plastic or viscoelastic deformations.

The stack of HAP platelets shown in Figure 3A,B is presumably caused by the shear stress τ_rz_ around podosomes (Figure 6D), provided that the HAP platelets are oriented parallel to the surface. Under this condition, the surface shear stress causes extended viscoelastic or plastic flow of the extrafibrillar matrix (the glue layer between the mineralized fibrils^42^). Together with the lytic activities in the resorption lacuna, also segments of the collagen triple helices can be destroyed. For the calculation, these data were derived from a detailed analysis of the anisotropic elastic constants of lamellar bone with plywood structure.^43^

### Degradation Caused by Podosome Assemblies

**Figure 7:**
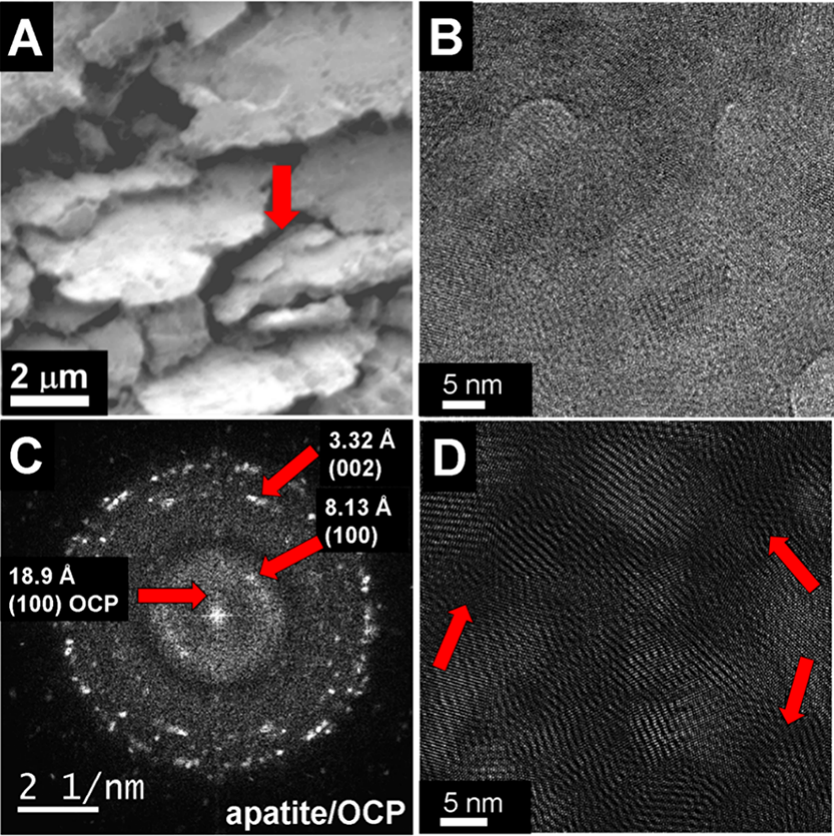
(A) The surface-sensitive secondary image of scanning electron microscopy (SEM) displays the presence of “intermediate” HAP plates (about 500 nm x 1000 nm in size) with already disturbed shape (marked by the arrow) in the “big plates” of HAP shown in Figure 5. (B) High-resolution TEM image of this area, see also region 3 of Figure 4. (C) The FFT image clearly shows the presence of HAP and octacalcium phosphate OCP. (D) The Fourier-filtered image shows crystalline regions with accentuated HAP lattice visible as bright stripes. The region between the nanocrystalline crystal plates is amorphous or strongly disturbed, see red arrows.

From the distribution of pits in Figures 2C, 4E,G, and 5, their average distance is estimated as 600–700 nm. The extension of the calculated first principal stress in Figure 6B (right) suggests that the induced stress covers almost the whole surface region available per podosome. The structure is stabilized by an increase in the density of dorsal actin cables as experimentally demonstrated by Luxenburg *et al*.^39^ The observed pit chains in Figures 2C, 4E, 5E–H, and 7 could be degradation traces of such cluster-like structures.

The “big plates” of HAP shown in Figure 5 result from high concentrations of Ca ions as well as ucOC. As mentioned already in connection with Figure 3B,C the solitary HAP platelets are larger than HAP platelets in mineralized healthy bone. It means that, in osteoporosis of elderly men, inhibition of platelet growth is reduced by impaired osteoblast genesis in an “aged” bone microenvironment. A possible explanation of this phenomenon could be a change of noncollagenous proteins (NCPs) produced by osteoblasts. Compared to healthy bone, a significant change of the structure of osteocalcin (OC) has been observed in osteoporotic bone.^44−47^ OC is the most abundant noncollagenous protein (NCP) in the extracellular matrix. Together with osteopontin (OPN), OC influences primarily the structure and mechanical behavior of the bone matrix.^45,46^ OC contains 46–50 amino acids, depending on the species. Two antiparallel α-helices, the “Gla helix” (α1) and the “Asp-Glu-helix” (α2), are framed by β-sheet structures. The carboxylated Gla proteins are small proteins, including γ-carboxyglutamic acid (Gla) with its two-fold negatively charged residue. It offers a perfect binding site for Ca^2+^. Because of three Gla residues of the α1-helix and the Asp-Glu residue of the α2-helix, carboxylated osteocalcin (cOC) has a strong binding efficiency to Ca^2+^ ions. This form is needed to stabilize Ca ions in solution.^48^

The synthesis of OC by osteoblasts is vitamin D- and vitamin K-dependent.^44^ Vitamin K is necessary for the posttranslational γ-carboxylation of glutamic acid residues. In connection with the growth of HAP minerals, two different forms of the total OC (tOC) have to be distinguished, the carboxylated OC (cOC) and the undercarboxylated OC (ucOC).^49^ As shown by Hoang *et al*.,^50^ the three Gla residues and the Asp residue of cOC can coordinate five Ca^2+^ ions on the prism face (100) and also on the secondary prism face (110) of HAP, which leads to highly regular HAP nanoplatelets in the mineralized collagen fibrils. However, the low affinity of ucOC to calcium ions causes a reduced bone quality (Hauschka *et al.*).^46^

The functionality of carboxylated osteocalcin (cOC) in mineralization has been interpreted as a process-directing agent for the intrafibrillar nucleation of HAP crystals in the type I collagen macrofibrils.^32,51,52^ The collagen microfibril can be regarded as the structure-directing agent.^10^ Particular motifs of amino acids initiate the crystallization of HAP (*c*-axis parallel to the fibril orientation). This assumption is supported by atomistic computer simulations of the interaction of the calcium and phosphate ions with a model molecule built of three polypeptide strands (glycin-proline-hydroxyproline) arranged in a triple-helical structure. (Gly-Pro-Hyp) is the dominating motif of amino acids in the tropocollagen filament.^53^ Additionally, cOC induces epitactic growth of OCP on HAP.^32^

There are various reports concerning the age-related change of the content of tOC and the ratio of cOC and ucOC in the serum of elderly population:

(i) increase in tOC reflects an increase in bone remodeling rate of elderly population associated with age-related bone loss,^7^
(ii) increase in ucOC caused by vitamin K deficiency in elderly population,^7^
(iii) ucOC may affect more the bone quality than the bone mineral density (BMD) in women over 60 years,^7^
(iv) increased content of serum ucOC is connected with an increasing hip fracture risk.^54^

The “big HAP plates” shown in Figure 7A. have been formed obviously under vitamin K deficiency. The resorption compartment was acidified by cytoplasmic H^+^ ions generated by cytoplasmic carbonic anhydrase from CO_2_ and H_2_O.^55,56^ The ions were transported through the ruffled border membrane by vacuolar H^+^-adenosine triphosphatase (H^+^-ATPase). Under these conditions (pH about 4.5), a high concentration of Ca^2+^ ions is produced by dissolution of HAP nanocrystals in the resorption compartment. Furthermore, the collagen filaments were at least partially degraded by the lysosomal proteolytic enzyme cathepsin K.^57^ Cathepsin K is expressed in the osteoclasts. It has to be assumed that there was a deficit of cOC in the osteoporotic bone.

The high-resolution images of the “big HAP plate” in Figure 7B,D and the Fast Fourier transform (FFT) image in Figure 7C show that the “big HAP plates” are nanocomposites consisting of randomly distributed HAP nanocrystals (characteristic size of about 3–5 nm) and a second amorphous calcium phosphate (ACP) phase. Obviously, the nucleation and random growth of the HAP nanoparticles are possible due to the nonsufficient concentration of the structure directing cOC. These nanocomposites possess a high similarity to calcium phosphate nanophases precipitated in supersaturated solutions with Ca^2+^, PO_4_^3–^, and OH^–^ ions (He *et al.*(58)). In this study, He *et al.* have shown that HAP nanocrystals (3–6 nm) heterogeneously nucleated on ACP. In a further step, those nanocrystals were grown by ACP dissolution–HAP reprecipitation followed by HAP self-assembly to a randomly aggregated nanocrystalline layer. Such a transition of ACP to HAP nanocrystals has recently been observed also by Montes-Hernandez and Renard.^59^

Similar studies with osteocalcin knockout mice (OC^–/–^ mice) have been performed by Moriishi *et al.*.^60^ They have shown that the deficit of cOC leads to a significant change of the orientation of the HAP nanocrystals in the mineralized collagen fibrils in comparison to wild-type mice. The bone mineral density (BMD) was similar between female wild-type and OC^–/–^ mice throughout the length of the femur at 9 months of age. The preferential orientation degree of the HAP *c*-axis, parallel to the bone longitudinal axis, was markedly lower in OC^–/–^ femurs than in the wild-type. Young’s modulus positively correlated with the HAP *c*-axis orientation degree, but not with BMD or the orientation of collagen. A multiple regression analysis showed that Young’s modulus was strongly and solely influenced by the orientation of HAP, but not by that of collagen or BMD in male mice at 14 weeks of age.

The model of a disturbed orientation of the HAP *c*-axis due to a deficit cOC along mineralized collagen fibrils has been verified also for knockout rats by Daghma *et al.*.^61^ They have compared the structure of mineralized collagen I fibrils in ovariectomized rats (OVX) with control rats (sham) at 16 months of age. In the selected area electron diffractogram, the preferred orientation of the HAP crystal along the *c*-axis with the corresponding (002) reflection has been measured. Different to the well-oriented HAP crystals in the control rats (sham), the OVX rats possessed a disoriented distribution of the HAP nanoplatelets caused by the deficit of process-directing cOC for their intrafibrillar nucleation. Similar results are shown in Suppl. Fig. S5. At healthy rat bone (Figure S5a–h), we find no degradation traces in opposite to the osteoporotic case, where the same thinned and degraded edge (Figures S5i,j) was observed as in the case of the human sample (Figure 1). Also, micro-cracks appear, see overview images Figures S5i,j at the top right and zoomed region in Figure S5k. The more misaligned, random orientation of apatite is demonstrated in the electron diffraction (Figure S5l).

In the bone matrix, the “big HAP plates” can be attacked by other osteoclasts, causing the new podosome traces visible in Figure 5E–H. The stacks of the “big HAP plates” represent critical defects in the osteoporotic bone. In comparison with individual HAP nanocrystals, the stress intensity caused by “big HAP plates” increases by a factor of about 10 under the assumption of equal external load. Furthermore, in the osteoporotic bone, the fracture toughness is significantly diminished for the propagating macrocrack. The macrocrack propagation is controlled by the crack resistance along the interface between the mineralized microfibrils.^62,63^ The stability of this extrafibrillar matrix is governed by the disruption of calcium-mediated ionic bonds between the long and irregular chains of carboxylated osteocalcin cOC and osteopontin OPN molecules constituting this matrix.^63^ In a study using bone tissue from genetic-deficient mice lacking cOC and/or OPN, Nikel *et al.* have shown by creep and fatigue tests that, in the absence of breakable bonds, the extrafibrillar matrix has significantly lower capacity for energy dissipation.^64^ At the same system, Poundarik *et al.*(65) have observed that the cOC-OPN adduct in bone allows it to resist fracture, and the loss of one or both proteins results in a loss of fracture toughness of similar size.

In conclusion, invasive podosomes and the enhanced formation of ucOC in a partially degraded collagen matrix under the conditions of a senile osteoporosis caused by vitamin K deficiency in elderly population are essential for defect formation in osteoporotic bone due to (i) the accumulation of nanosized microcracks induced by the local stress field of oscillating podosomes; (ii) the deficit of cOC, which leads to a significant change of the orientation of the HAP nanocrystals in the mineralized collagen fibrils; and (iii) the accelerated growth of “big HAP plates” initiated by the enhanced formation of ucOC.

## Conclusions

The degradation of aged osteoporotic human trabecular bone is caused by a lack of osteoblast cell activity and thus unbalanced osteoclast activity, leading to depletion of the bone structure. Our TEM and SEM analysis revealed heavy degradation of osteoporotic bone. At high resolution, the fragmented bone pieces show pit-like traces on the migration path when the osteoclast has stopped bone resorption and migrates on ECM surfaces. Such a pattern is formed when the sealing zone has a ring-like shape. The degradation of a flat HAP layer along the migration path results in partially free lying collagen fibrils. Collagen fibrils disintegrate within the massive bone pieces due to podosome activity. Instead of collagen fibrils, a fine gelatinous network of denaturized collagen was observed.

Free apatite nanoplatelets are formed in the degradation zone of single podosomes. These platelets are 2–3 times larger than expected for normal bone due to the larger content of undercarboxylated glutamic acid residues of osteocalcin, being responsible for accelerated platelet growth. Eventually, several micron-sized HAP sheets are formed.

The invasive podosomes and the enhanced formation of ucOC in a partially degraded collagen matrix under the conditions of a senile osteoporosis caused by vitamin K deficiency in elderly population are essential for defect formation in osteoporotic bone due to (i) the accumulation of nanosized microcracks induced by the local stress field of oscillating podosomes; (ii) the deficit of cOC, which leads to a significant change of the orientation of the HAP nanocrystals in the mineralized collagen fibrils; and (iii) the accelerated growth of “big HAP plates” initiated by the enhanced formation of ucOC.

## Methods

Full details of the sample preparation and sample characterization techniques used are presented in the Supporting Information.

## Ethical Approval and Informed Consent

This study was performed in full compliance with the institutional German laws. All experiments were approved by the ethical commission of the local governmental institution (“Regierungspräsidium Giessen”, permit number: 89/2009). See also the Methods section.

Due to ethical reasons, the access to healthy human bone is restricted; thus, we could not obtain such a sample. Nevertheless, the authors have several publications about healthy bone of rats within the frame of the past project of a multicenter study and part of the Collaborating Research Center funding program SFB/Transregio 79 of the German Research Foundation DFG together with the Experimental Trauma Surgery of the Justus-Liebig University, Giessen, Germany from 2010 to 2014, see also refs (32, 61). Furthermore, in Suppl. Fig. S5, new experimental data are shown for healthy and ovariectomized bone of rat to evaluate the influence of the deficit of carboxylated osteocalcin on the degree of *c*-axis orientation in mineralized collagen fibrils.

## Supporting Information Available

(Figure S1) Light microscopy of semithin cut of central part of femoral head; (Figure S2) zoom series of podosome track; (Figure S3) energy-filtered TEM (EFTEM) of the isolated brittle bone island; (Figure S4) calculation of the stress distribution around a podosome; (Figure S5) healthy rat bone from femur (trabecular) and spine (cortical) and osteoporotic form spine (cortical); (Methods) sample preparation, SEM and TEM, energy-filtered TEM, and stress modeling (PDF)

## References

[ref1] Bailey A. J.; Wotton S. F.; Sims T. J.; Thompson P. W. Biochemical changes in the collagen of human osteoporotic bone matrix. Connect. Tissue Res. 1993, 29, 119–132. 10.3109/03008209309014239. 8403893

[ref2] Yu B.; Wang C.-Y. Osteoporosis: The Result of an ’Aged’ Bone Microenvironment. Trends Mol. Med. 2016, 22, 641–644. 10.1016/j.molmed.2016.06.002. 27354328 PMC4969144

[ref3] Ruffoni D.; Fratzl P.; Roschger P.; Klaushofer K.; Weinkamer R. The bone mineralization density distribution as a fingerprint of the mineralization process. Bone 2007, 40, 1308–1319. 10.1016/j.bone.2007.01.012. 17337263

[ref4] Shen Y.; Zhang Z.-M.; Jiang S.-D.; Jiang L.-S.; Dai L.-Y. Postmenopausal women with osteoarthritis and osteoporosis show different ultrastructural. characteristics of trabecular bone of the femoral head. BMC Musculoskelet. Disord. 2009, 10, 1–12. 10.1186/1471-2474-10-35. 19356253 PMC2674588

[ref5] Sahar N. D.; Hong S.-I.; Kohn D. H. Micro- and nano-structural analyses of damage in bone. Micron 2005, 36, 617–629. 10.1016/j.micron.2005.07.006. 16169739

[ref6] Tzaphlidou M. Bone Architecture: Collagen Structure and Calcium/Phosphorus Maps. J. Biol. Phys. 2008, 34, 39–49. 10.1007/s10867-008-9115-y. 19669491 PMC2577747

[ref7] Liu G.; Peacock M. Age-related changes in serum undercarboxylated osteocalcin and its relationships with bone density, bone quality and hip fracture. Calcif. Tissue Int. 1998, 62, 286–289. 10.1007/s002239900432. 9504950

[ref8] Ozasa R.; Ishimoto T.; Miyabe S.; Hashimoto J.; Hirao M.; Yoshikawa H.; Nakano T. Osteoporosis Changes Collagen/Apatite Orientation and Young’s Modulus in Vertebral Cortical Bone of Rat. Calcif. Tissue Int. 2019, 104, 449–460. 10.1007/s00223-018-0508-z. 30588540

[ref9] Rubin M. A.; Jasiuk I.; Taylor J.; Rubin J.; Ganey T.; Apkarian R. P. TEM analysis of the nanostructure of normal and osteoporotic human trabecular bone. Bone 2003, 33, 270–282. 10.1016/s8756-3282(03)00194-7. 13678767

[ref10] Nudelman F.; Pieterse K.; George A.; Bomans P. H. H.; Friedrich H.; Brylka L. J.; Hilbers P. A. J.; de With G.; Sommerdijk N. A. J. M. The role of collagen in bone apatite formation in the presence of hydroxyapatite nucleation inhibitors. Nat. Mater. 2010, 9, 1004–1009. 10.1038/NMAT2875. 20972429 PMC3084378

[ref11] Li Z.; Lu W. W.; Deng L.; Chiu P. K. Y.; Fang D.; Lam R. W. M.; Leong J. C. Y.; Luk K. D. K. The morphology and lattice structure of bone crystal after strontium treatment in goats. J. Bone Miner. Metab. 2010, 28, 25–34. 10.1007/s00774-009-0109-z. 19603246

[ref12] Cummings S. R.; Melton L. J. Epidemiology and outcomes of osteoporotic fractures. Lancet 2002, 359, 1761–1767. 10.1016/S0140-6736(02)08657-9. 12049882

[ref13] Riggs B. L.; Melton L. J. III The worldwide problem of osteoporosis: insights afforded by epidemiology. Bone 1995, 17, 505S–S511. 10.1016/8756-3282(95)00258-4. 8573428

[ref14] Arcos D.; Boccaccini A. R.; Bohner M.; Díez-Pérez A.; Epple M.; Gómez-Barrena E.; Herrera A.; Planell J. A.; Rodríguez-Mañas L.; Vallet-Regí M. The relevance of biomaterials to the prevention and treatment of osteoporosis. Acta Biomater. 2014, 10, 1793–1805. 10.1016/j.actbio.2014.01.004. 24418434

[ref15] McNamara L. M. Perspective on post-menopausal osteoporosis: establishing an interdisciplinary understanding of the sequence of events from the molecular level to whole bone fractures. J. R. Soc., Interface 2010, 7, 353–372. 10.1098/rsif.2009.0282. 19846441 PMC2842799

[ref16] Zoehrer R.; Roschger P.; Paschalis E. P.; Hofstaetter J. G.; Durchschlag E.; Fratzl P.; Phipps R.; Klaushofer K. Effects of 3- and 5-year treatment with risedronate on bone mineralization density distribution in triple biopsies of the iliac crest in postmenopausal women. J. Bone Miner. Res. 2006, 21, 1106–1112. 10.1359/jbmr.060401. 16813531

[ref17] Jakob F. Neue Targets in der Osteoporosetherapie. Deutsche medizinische Wochenschrift (1946) 2011, 136, 898–903. 10.1055/s-0031-1275826. 21523643

[ref18] Rachner T. D.; Khosla S.; Hofbauer L. C. Osteoporosis: now and the future. Lancet 2011, 377, 1276–1287. 10.1016/S0140-6736(10)62349-5. 21450337 PMC3555696

[ref19] Rodríguez J. P.; Montecinos L.; Ríos S.; Reyes P.; Martínez J. Mesenchymal stem cells from osteoporotic patients produce a type I collagen-deficient extracellular matrix favoring adipogenic differentiation. J. Cell. Biochem. 2000, 79, 557–565. 10.1002/1097-4644(20001215)79:4<557:aid-jcb40>3.0.co;2-h. 10996846

[ref20] Saito M.; Marumo K. Collagen cross-links as a determinant of bone quality: a possible explanation for bone fragility in aging, osteoporosis, and diabetes mellitus. Osteoporos. Int. 2010, 21, 195–214. 10.1007/s00198-009-1066-z. 19760059

[ref21] Henss A.; Rohnke M.; El Khassawna T.; Govindarajan P.; Schlewitz G.; Heiss C.; Janek J. Applicability of ToF-SIMS for monitoring compositional changes in bone in a long-term animal model. J. R. Soc., Interface 2013, 10, 20130332 10.1098/rsif.2013.0332. 23864501 PMC3730683

[ref22] Weiner S.; Traub W.; Wagner H. D. Lamellar Bone: Structure -Function Relations. J. Struct. Biol. 1999, 126, 241–255. 10.1006/jsbi.1999.4107. 10475685

[ref23] Trump B. F.; Schmuckler E. A.; Benditt E. P. A method for staining epoxy sections for light microscopy. J. Ultrastruct. Res. 1961, 5, 343–348. 10.1016/S0022-5320(61)80011-7. 13922704

[ref24] Ito S.; Winchester R. J. The fine structure of the gastric mucosa in the bat. J. Cell Biol. 1963, 16, 541–577. 10.1083/jcb.16.3.541. 13957001 PMC2106229

[ref25] Akisaka T.; Yoshida A. Ultrastructural analysis of apatite-degrading capability of extendedinvasive podosomes in resorbing osteoclasts. Micron 2016, 88, 37–47. 10.1016/j.micron.2016.05.006. 27323283

[ref26] Le N.; Xue M.; Castelnoble L. A.; Jackson C. J. The dual personalities of matrix metalloproteinases in inflammation. Front. Biosci. 2007, 12, 1475–1487. 10.2741/2161. 17127395

[ref27] Liang H. P. H.; Xu J.; Xue M.; Jackson C. Matrix metalloproteinases in bone development and pathology: current knowledge and potential clinical utility. Metalloproteinases Med. 2016, Volume 3, 93–102. 10.2147/MNM.S92187.

[ref28] Linder S.; Wiesner C. Tools of the trade: podosomes as multipurpose organelles of monocytic cells. Cell. Mol. Life Sci. 2015, 72, 121–135. 10.1007/s00018-014-1731-z. 25300510 PMC11113205

[ref29] Akisaka T.; Yoshida H.; Suzuki R. The ruffled border and attachment regions of the apposing membrane of resorbing osteoclasts as visualized from the cytoplasmic face of the membrane. J. Electron Microsc. 2006, 55, 53–61. 10.1093/jmicro/dfl012. 16775216

[ref30] Georgess D.; Machuca-Ggayet I.; Blangy A.; Jurdic P. Podosme organization drives osteodclast-mediated bone resorption. Cell Adhes. Migr. 2014, 8, 192–204. 10.4161/cam.27840. PMC4198343 24714644

[ref31] Takito J.; Inoue S.; Nakamura M. The Sealing Zone in Osteoclasts: A Self-Organized Structure on the Bone. Int. J. Mol. Sci. 2018, 19, 984–997. 10.3390/ijms19040984. PMC5979552 29587415

[ref32] Simon P.; Grüner D.; Worch H.; Pompe W.; Lichte H.; El Khassawna T.; Heiss C.; Wenisch S.; Kniep R. First evidence of octacalcium phosphate@osteocalcin nanocomplex as skeletal bone component directing collagen triple–helix nanofibril mineralization. Sci. Rep. 2018, 8, 13696 10.1038/s41598-018-31983-5. 30209287 PMC6135843

[ref33] Rubin M. A.; Jasiuk I. The TEM characterization of the lamellar structure of osteoporotic human trabecular bone. Micron 2005, 36, 653–664. 10.1016/j.micron.2005.07.010. 16198582

[ref34] Gentzsch C.; Delling G.; Kaiser E. Microstructural classification of resorption lacunae and perforations in human proximal femora. Calcif. Tissue Int. 2003, 72, 698–709. 10.1007/s00223-002-2020-7. 14562999

[ref35] Schachtner H.; Calaminus S. D. J.; Thomas S. G.; Machesky L. M. Podosomes in adhesion, migration, mechanosensing and matrix remodeling. Cytoskeleton 2013, 70, 572–589. 10.1002/cm.21119. 23804547

[ref36] Collin O.; Na S.; Chowdhury F.; Hong M.; Shin M. E.; Wang F.; Wang N. Self-organized podosomes are dynamic mechanosensors. Curr. Biol. 2008, 18, 1288–1294. 10.1016/j.cub.2008.07.046. 18760605 PMC2605691

[ref37] Akisaka T.; Yoshida A. Ultrastructural analysis of apatite-degrading capability of extended invasive podosomes in resorbing osteoclasts. Micron 2016, 88, 37–47. 10.1016/j.micron.2016.05.006. 27323283

[ref38] van den Dries K.; Nahidiazar L.; Slotman J. A.; Meddens M. B. M.; Pandzic E.; Joosten B.; Ansems M.; Schouwstra J.; Meijer A.; Steen R.; Wijers M.; Fransen J.; Houtsmuller A. B.; Wiseman P. W.; Jalink K.; Cambi A. Modular actin nano-architecture enables podosome protrusion and mechanosensing. Nat. Commun. 2019, 10, 5171 10.1038/s41467-019-13123-3. 31729386 PMC6858452

[ref39] Luxenburg C.; Geblinger D.; Klein E.; Anderson K.; Hanein D.; Geiger B.; Addadi L. The Architecture of the Adhesive Apparatus of Cultured Osteoclasts: From Podosome Formation to Sealing Zone Assembly. PLoS One 2007, 2, e179 10.1371/journal.pone.0000179. 17264882 PMC1779809

[ref40] Merrild D. M.; Pirapaharan D. C.; Andreasen C. M.; Kjærsgaard-Andersen P.; Møller A. M.; Ding M.; Delaisse J.-M.; Søe K. Pit- and trench-forming osteoclasts: A distinction that matters. Bone Res. 2015, 3, 15032 10.1038/boneres.2015.32. 26664853 PMC4665108

[ref41] Labernadie A.; Bouissou A.; Delobelle P.; Balor S.; Voituriez R.; Proag A.; Fourquaux I.; Thibault C.; Vieu C.; Poincloux R.; Charrière G. M.; Maridonneau-Parini I. Protrusion force microscopy reveals oscillatory force generation and mechanosensing activity of human macrophage podosomes. Nat. Commun. 2014, 5, 5343 10.1038/ncomms6343. 25385672

[ref42] Gupta H. S.; Fratzl P.; Kerschnitzki M.; Benecke G.; Wagermaier W.; Kirchner H. O. K. Evidence for an elementary process in bone plasticity with an activation enthalpy of 1 eV. J. R. Soc., Interface 2007, 4, 277–282. 10.1098/rsif.2006.0172. 17251154 PMC2220070

[ref43] Carnelli D.; Vena P.; Dao M.; Ortiz C.; Contro R. Orientation and size-dependent mechanical modulation within individual secondary osteons in cortical bone tissue secondary osteons in cortical bone tissue. J. R. Soc., Interface 2013, 10, 1–12. 10.1098/rsif.2012.0953. PMC3627101 23389895

[ref44] Fusaro M.; Cianciolo G.; Brandi M. L.; Ferrari S.; Nickolas T. L.; Tripepi G.; Plebani M.; Zaninotto M.; Iervasi G.; La Manna G.; Gallieni M.; Vettore R.; Aghi A.; Gasperoni L.; Giannini S.; Sella S.; Cheung A. Vitamin K and Osteoporosis. Nutrients 2020, 12, 3625–3637. 10.3390/nu12123625. PMC7760385 33255760

[ref45] Hunter G. K.; Hauschka P. V.; Poole A. R.; Rosenberg L. C.; Goldberg H. A. Nucleation and inhibition of hydroxyapatite formation by mineralized tissue proteins. Biochem. J. 1996, 317, 59–64. 10.1042/bj3170059. 8694787 PMC1217486

[ref46] Hauschka P. V.; Lian J. B.; Cole D. E.; Gundberg C. M. Osteocalcin and matrix Gla protein: Vitamin K-dependent proteins in bone. Physiol. Rev. 1989, 69, 990–1047. 10.1152/physrev.1989.69.3.990. 2664828

[ref47] Berezovska O.; Yildirim G.; Budell W. C.; Yagerman S.; Pidhaynyy B.; Bastien C.; van der Meulen M. C. H.; Dowd T. L. Osteocalcin affects bone mineral and mechanical properties in female mice. Bone 2019, 128, 115031 10.1016/j.bone.2019.08.004. 31401301 PMC8243730

[ref48] Ducy P.; Desbois C.; Boyce B.; Pinero G.; Story B.; Dunstan C.; Smith E.; Bonadio J.; Goldstein S.; Gundberg C.; Bradley A.; Karsenty G. Increased bone formation in osteocalcin-deficient mice. Nature 1996, 382, 448–452. 10.1038/382448a0. 8684484

[ref49] Neve A.; Corrado A.; Cantatore F. P. Osteocalcin: Skeletal and extra-skeletal effects. J. Cell. Physiol. 2013, 228, 1149–1153. 10.1002/jcp.24278. 23139068

[ref50] Hoang Q. Q.; Sicheri F.; Howard A. J.; Yang D. S. C. Bone recognition mechanism of porcine osteocalcin from crystal structure. Nature 2003, 425, 977–980. 10.1038/nature02079. 14586470

[ref51] Chen L.; Jaquet R.; Lowder E.; Landis W. J. Refinement of collagen mineral interaction: A possible role for osteocalcin in apatite crystal nucleation, growth and development. Bone 2015, 71, 7–16. 10.1016/j.bone.2014.09.021. 25284158

[ref52] Olszta M. J.; Cheng X.; Jee S. S.; Kumar R.; Kim Y.-Y.; Kaufman M. J.; Douglas E. P.; Gower L. B. Bone structure and formation: A new perspective. Mater. Sci. Eng. 2007, 58, 77–116. 10.1016/j.mser.2007.05.001.

[ref53] Kawska A.; Hochrein O.; Brickmann J.; Kniep R.; Zahn D. The nucleation mechanism of fluorapatite–collagen composites: ion association and motif control by collagen proteins. Angew. Chem., Int. Ed. 2008, 47, 4982–4985. 10.1002/anie.200800908. 18496823

[ref54] Vergnaud P.; Garnero P.; Meunier P. J.; Bréart G.; Kamihagi K.; Delmas P. D. Undercarboxylated Osteocalcin Measured with a Specific Immunoassay Predicts Hip Fracture in Elderly Women: The EPIDOS Study. J. Clin. Endocrinol. Metab. 1997, 82, 719–724. 10.1210/jcem.82.3.3805. 9062471

[ref55] Silver I. A.; Murrills R. J.; Etherington D. J. Microelectrode studies on the acid microenvironment beneath adherent macrophages and osteoclasts. Exp. Cell Res. 1988, 175, 266–276. 10.1016/0014-4827(88)90191-7. 3360056

[ref56] Blair H. C.; Kahn A. J.; Crouch E. C.; Jeffrey J. J.; Teitelbaum S. L. Isolated osteoclasts resorb the organic and inorganic components of bone. J. Cell. Biol. 1986, 102, 1164–1172. 10.1083/jcb.102.4.1164. 3457013 PMC2114153

[ref57] Drake F. H.; Dodds R. A.; James I. E.; Connor J. R.; Debouck C.; Richardson S.; Lee-Rykaczewski E.; Coleman L.; Rieman D.; Barthlow R.; Hastings G.; Gowen M. Cathepsin K, but Not Cathepsins B, L, or S, Is Abundantly Expressed in Human Osteoclasts. J. Biol. Chem. 1996, 271, 12511–12516. 10.1074/jbc.271.21.12511. 8647859

[ref58] He K.; Sawczy M.; Liu C.; Yuan Y.; Song B.; Delvanayagam R.; Nie A.; Hu X.; Dravid V. P.; Lu J.; Sukotjo C.; Lu Y.; Kral P.; Shokuhfar T.; Shahbazian-Yassar R. Revealing nanoscale mineralization pathways of hydroxyapatite using in situ liquid cell transmission electron microscopy. Sci. Adv. 2020, 6, 1–11. 10.1126/sciadv.aaz7524. PMC7673812 33208378

[ref59] Montes-Hernandez G.; Renard F. Nucleation of Brushite and Hydroxyapatite from Amorphous Calcium Phosphate Phases Revealed by Dynamic In Situ Raman Spectroscopy. Cryst. Growth Des. 2016, 16, 7218–7230. 10.1021/acs.cgd.6b01406.

[ref60] Moriishi T.; Ozasa R.; Ishimoto T.; Nakano T.; Hasegawa T.; Miyazaki T. Osteocalcin is necessary for the alignment of apatite crystallites, but not glucose metabolism, testosterone synthesis, or muscle mass muscle mass metabolism, testosterone synthesis, or muscle mass. PLoS Genet. 2020, 16, e1008586 10.1371/journal.pgen.1008586. 32463816 PMC7255595

[ref61] Daghma D. E. S.; Malhan D.; Simon P.; Stötzel S.; Kern S.; Hassan F.; Lips K. S.; Heiss C.; Khassawna T. E. Computational segmentation of collagen fibers in bone matrix indicates bone quality in ovariectomized rat spine. J. Bone Miner. Metab. 2018, 36, 297–306. 10.1007/s00774-017-0844-5. 28589410

[ref62] Gupta H. S.; Seto J.; Wagermaier W.; Zaslansky P.; Boesecke P.; Fratzl P. Cooperative deformation of mineral and collagen in bone at the nanoscale. Proc. Natl. Acad. Sci. 2006, 17741–17746. 10.1073/pnas.0604237103. 17095608 PMC1635545

[ref63] Fratzl P.; Weinkamer R. Nature’s hierarchical materials. Prog. Mater. Sci. 2007, 52, 1263–1334. 10.1016/j.pmatsci.2007.06.001.

[ref64] Nikel O.; Poundarik A. A.; Bailey S.; Vashishth D. Structural role of Osteocalcin and Osteopontin in Energy Dissipation in Bone. J. Biomech. 2018, 80, 45–52. 10.1016/j.jbiomech.2018.08.014. 30205977 PMC6188842

[ref65] Poundarik A. A.; Diab T.; Sroga G. E.; Ural A.; Boskey A.; Gundberg C. M.; Vashishth D. Dilatational band formation in bone. Proc. Natl. Acad. Sci. 2012, 109, 19178–19183. 10.1073/pnas.1201513109. 23129653 PMC3511118

