# Supplementary material for: Podosome-Driven Defect Development in Lamellar Bone under the Conditions of Senile Osteoporosis Observed at the Nanometer Scale
Source: ACS Biomater Sci Eng. 2021 May 3;7(6):2255–67. doi: 10.1021/acsbiomaterials.0c01493 (PMC8290401; doi:10.1021/acsbiomaterials.0c01493)
Supplement: Supplementary file 1 — ab0c01493_si_001.pdf [file ab0c01493_si_001.pdf]

Supplementary information

Podosomes driven defect development in lamellar bone under conditions of senile osteoporosis observed at the nanometer scale

Paul Simon[1*], Wolfgang Pompe[2], Manfred Bobeth[2], Hartmut Worch[2], Rüdiger Kniep[1], Petr Formanek[3], Anne Hild[4], Sabine Wenisch[4], Elena Sturm[1,5]

[1] *Max-Planck-Institut für Chemische Physik fester Stoffe, Nöthnitzer Str. 40, 01187 Dresden, Germany*

[2] *Technical University of Dresden, Institute of Materials Science, 01069 Dresden, Germany*

[3] *Leibniz-Institut für Polymerforschung Dresden e.V., Hohe Straße 6, 01069 Dresden, Germany*

[4] *Clinical Anatomy, Clinic of Small Animals, Justus-Liebig-University, 35385 Giessen, Germany*

[5] *University of Konstanz, Physical Chemistry, POB 714, D-78457 Konstanz, Germany*

1. Figure S1	Light microscopy of semithin cut of central part of femoral head
2. Figure S2	Zoom series of podosome track
3. Figure S3	Energy filtered TEM (EFTEM) of isolated brittle bone island
4. Figure S4	Calculation of the stress distribution around a podosome
5. Figure S5	Healthy rat bone from femur (trabecular) and spine (cortical) and ovariectomized rat bone from spine (cortical)
6. Methods	Sample preparation, SEM and TEM, Energy Filtered TEM, Stress modeling

1. Figure S1

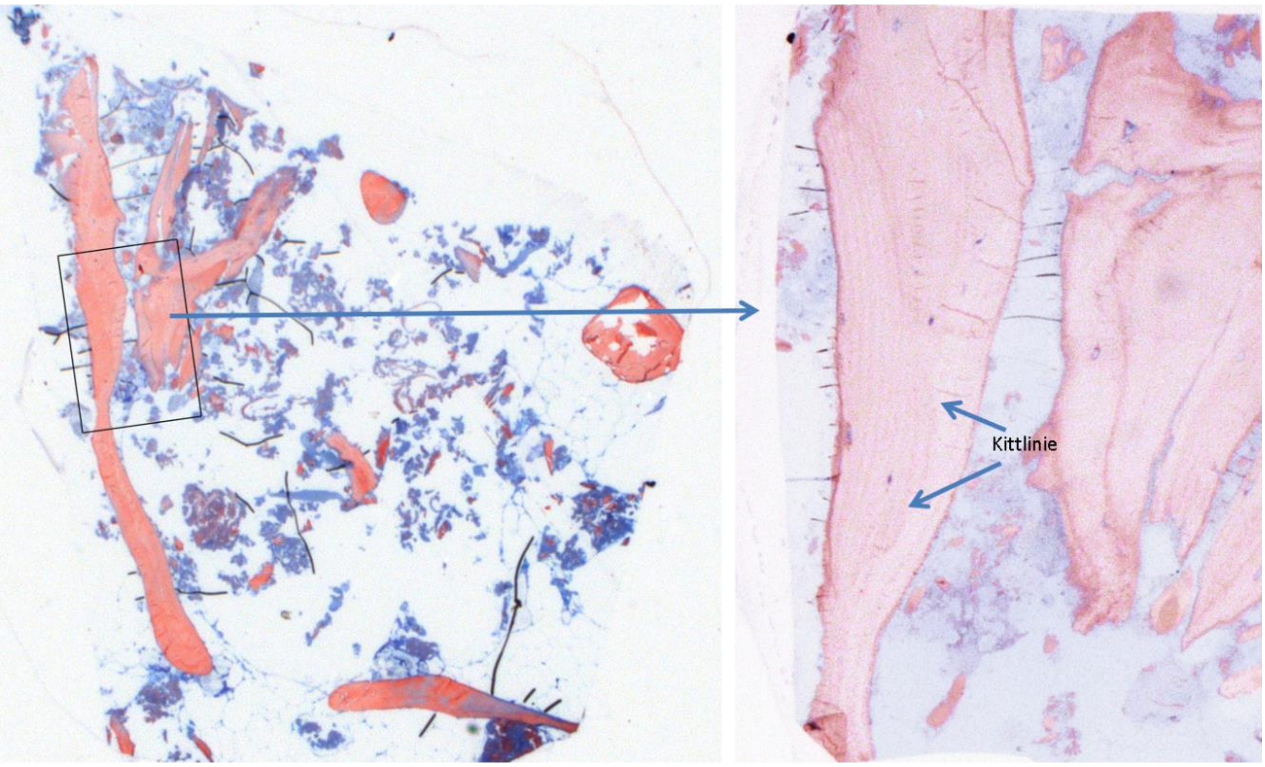

Supplementary Figure 1: *Left: Overview stained light microscopy image of central part of femur head showing trabecula. Right: Investigated region of interest, see also Fig. 1. Field of view on the left amounts about 2 mm, on the right ca. 0.4 mm (x-axis).*

2. Figure S2

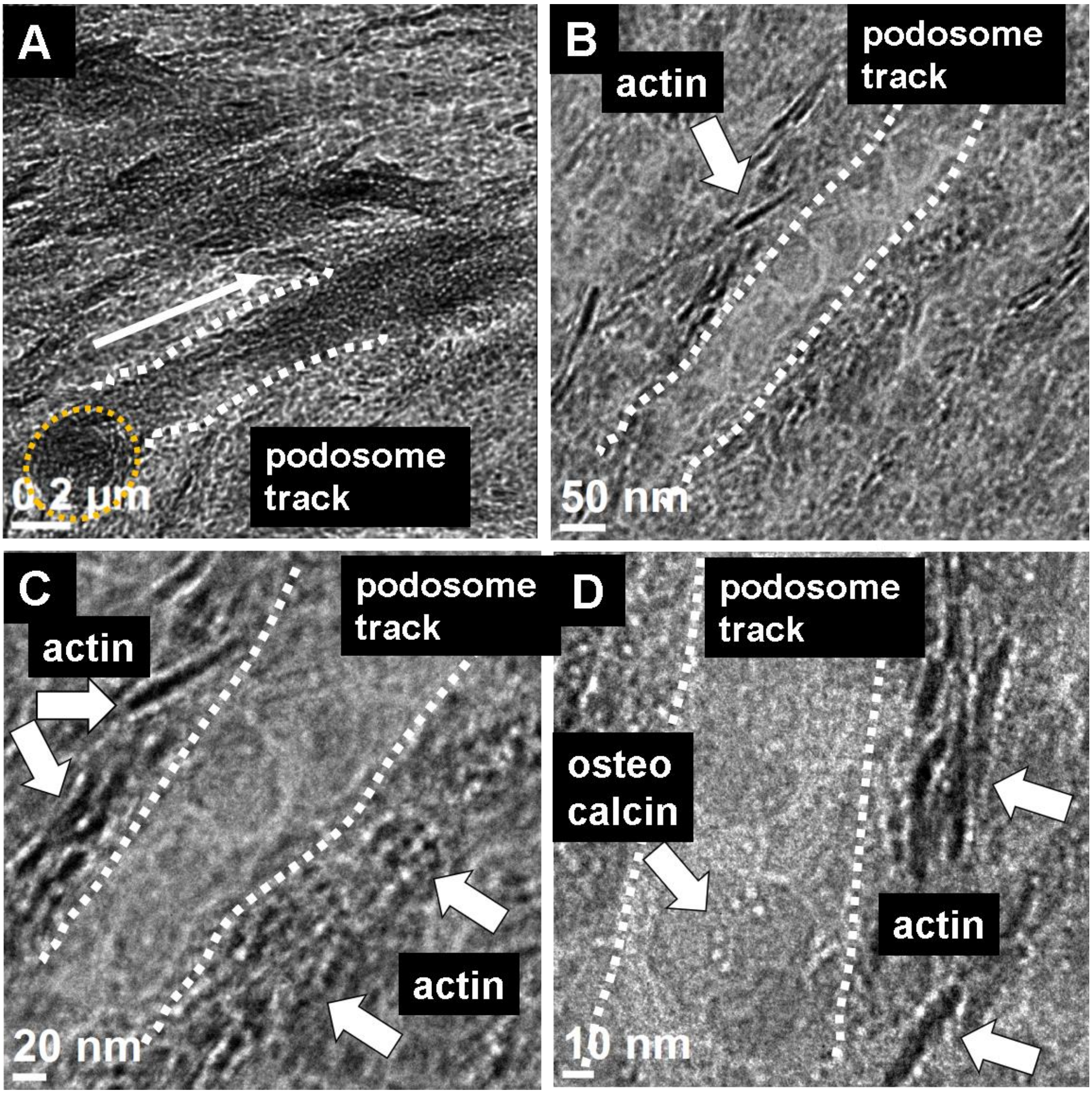

Supplementary Figure 2. *Podosome migration. (a) Track of podosome (yellow circle) movement, see white arrow. (b-d) Zoom series into track (see white dotted lines) where the surface material of the lamella is removed. Right and left remnant actin filaments are observed.*

3. Figure S3

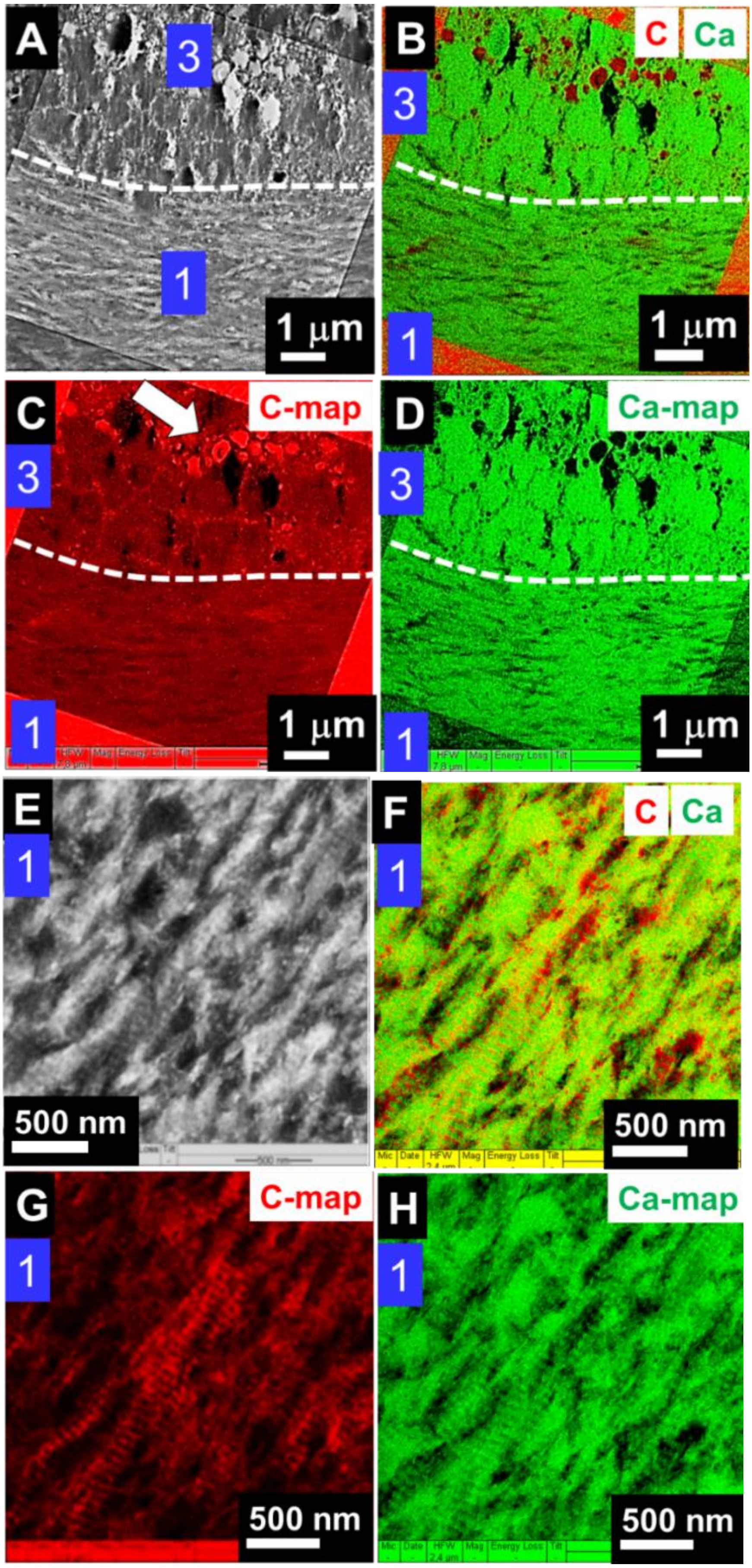
A
3
1
1 μm
B
C
Ca
3
1
1 μm
C
C-map
3
1
1 μm
D
Ca-map
3
1
1 μm
E
1
500 nm
F
C
Ca
1
500 nm
G
C-map
1
500 nm
H
Ca-map
1
500 nm

Supplementary Figure 3. *Energy filtered TEM (EFTEM) of isolated bone island shown in Figs. 4 and 5. Interface of region 3 (with significant lack of collagen fibrils) and etched region 1 (the most preserved structure). (a) Bright field image overview. (b) Combined carbon (red) and calcium (green) map reveals that in this region mainly HAP is present. The collagen content is strongly reduced. (c) Carbon map indicating only tiny amounts of organics (arrow, bright red), stemming from actin fibers or denaturated gelatinous collagen in the area 3. (d) Calcium map revealing a high content of HAP (green) in the region 3 as well as in region 1 where mineralized collagen fibrils are still present. (e) Enlarged bright field image of etched region 1 displaying collagen fibrils. (f) Combined carbon (red) and calcium map (green) of region 1. Collagen content (red) is strongly reduced, mainly present in the center. HAP (green) dominates. The Ca-distribution is inhomogeneous due to acidic depletion. (g) Carbon map of region 1 displaying remnants of collagen fibrils in the center. (h) Calcium map shows HAP mineral distribution. HAP is partially etched away (dark areas).*

4. Calculation of the stress distribution

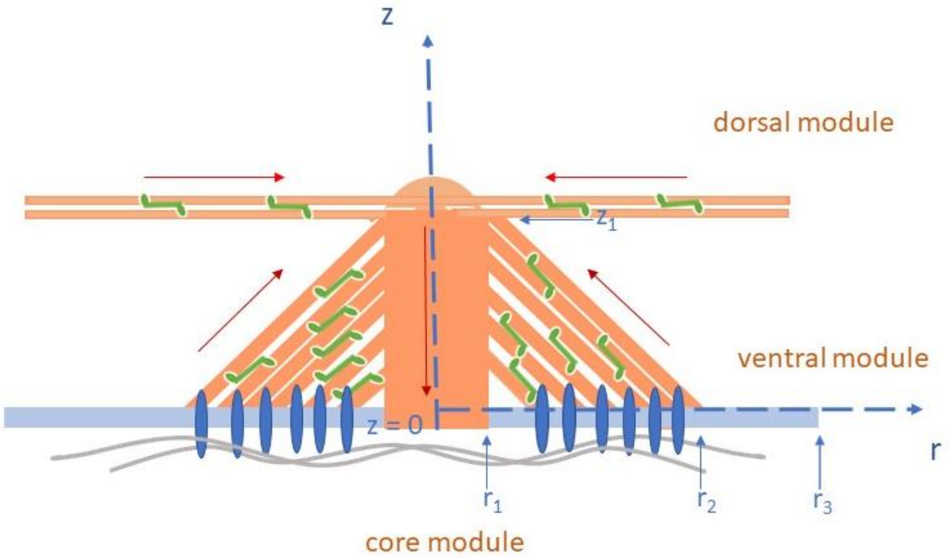

Supplementary Figure 4. Schematic model of the force equilibrium in a single podosome

Force equilibrium in *z*-direction requires

$F_c = - F_v \cdot \sin\varphi$ with $\tan\varphi = z_1/(r_2-r_1)$. (S1)

The corresponding average traction forces acting onto the lamella surface are given by

$t_z = F_c/\pi r_1^2$ for $0 < r \leq r_1$,

$t_z = F_v \cdot \sin\varphi/\pi\cdot(r_2^2-r_1^2)$ for $r_1 < r \leq r_2$,

$t_z = 0$ for $r_2 < r \leq r_3$

$t_r = 0$ for $0 < r \leq r_1$, (S2)

$t_r = - F_v \cdot \cos\varphi/\pi\cdot(r_3^2-r_2^2)$ for $r_1 < r \leq r_2$,

$t_r = 0$ for $r_2 < r \leq r_3$.

Furthermore, the boundary condition for the displacement

$u_r(r = r_4, z) = 0$ (S3)

has to be fulfilled.

In the calculations the following data have been used:

F_c= -150 nN, E_{eff} = 18 GPa, ν_{eff} = 0.35;

r_1 = 150 nm, r_2 = 250 nm, r_3 = 1500 nm;

for an individual podosome;

r_3 = 375 nm for a podosome located in a cluster;

z_1 = 600 nm.

Supplementary Figure 5.

TEM images of the mineralization pattern of the healthy rat (sham bone) femur and spine and osteoporotic ovariectomized rat spine.

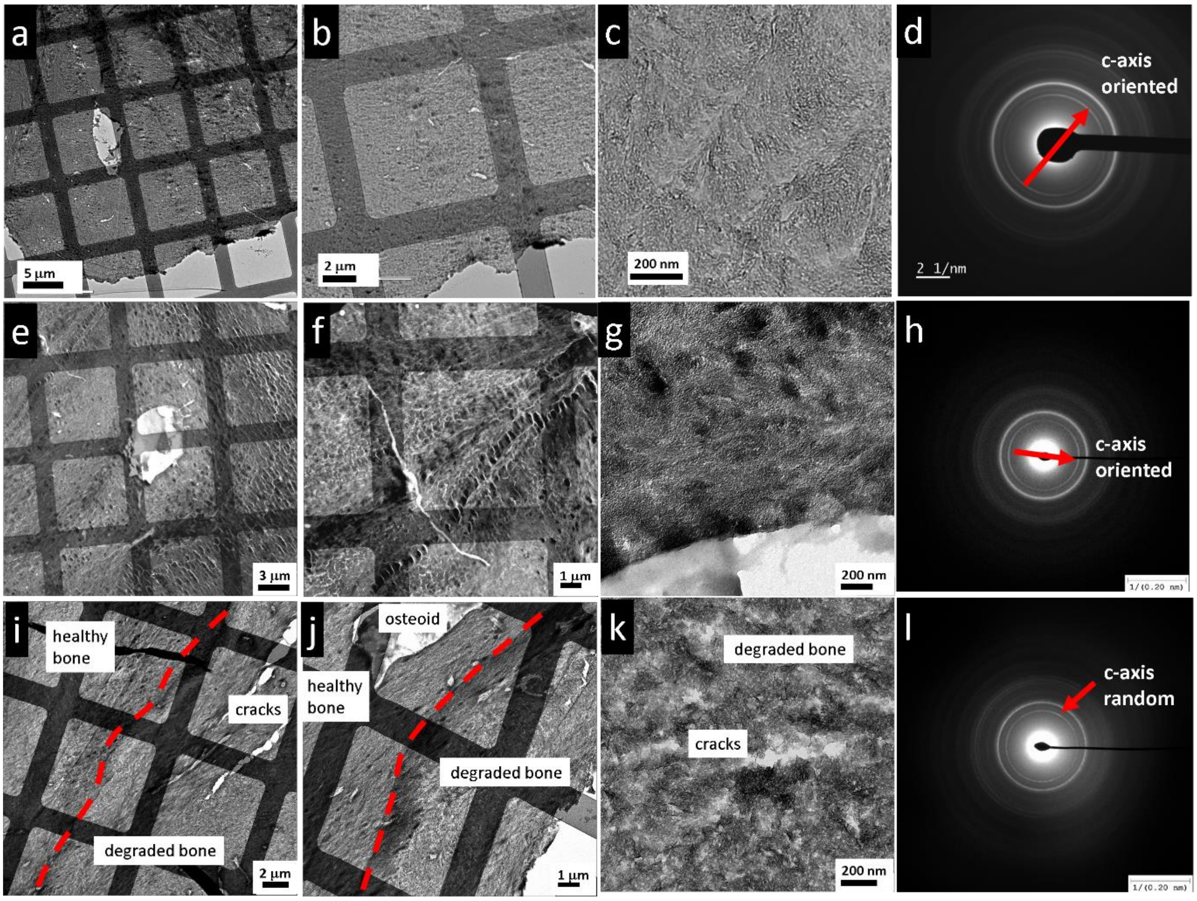

Supplementary Figure 5. TEM images of the mineralization pattern of the healthy rat (sham bone) femur (a-d) and spine (e-h), and osteoporotic ovariectomized rat spine (i-l). (a) Overview of healthy femur rat bone. (b-c) Zoom into edge reveals a regular structure without degradation processes. (d) Diffraction shows preferred orientation of apatite c-axis corresponding to (002) reflection. (e) Overview of thin cut of rat spine cortical bone with osteoid in the center. (f-g) Enlargement reveals a homogenous pattern without damages caused by osteoclast activity. (h) Diffraction proves the *c*-axis alignment of apatite along the collagen fibrils (red arrow) indicated by arc-like appearance of the (002) reflection. (i-k) Osteoporotic rat bone from the spine region. At the edge of the thin cut, degradation is well visible. It is manifested by a thinned region on the right and the presence of cracks. In the selected area electron diffractogram the preferred orientation of the HAP crystal along the *c*-axis with the corresponding (002) reflections is observed (d and h). Differently, in the ovariectomized bone the diagram (l) shows a (002) reflection (red arrow), which corresponds to a random distribution of the HAP nanoplatelets caused by the deficit of process-directing cOC for their intrafibrillar nucleation.

6. Methods

Sample preparation

Cancellous bone samples were removed from the central part of femoral head of a 67 years old male patient undergoing total hip arthroplasty because of fragility fracture of the femoral neck. The whole femoral head was received from surgery and cut in smaller pieces about 2x2x4 mm by a water-cooled diamond saw band for on the same day for TEM preparation. Until the cut, the pieces were stored in a polytetrafluorethylene (PTFE) box filled with 500 ml phosphate buffered saline at pH 7,2, which was sterilized in an autoclave. It was important to work under sterile conditions, since samples were also used for further molecular biologic experiments besides histological investigations. The saw table including saw blade were cleaned by 70% alcohol. The water for cooling the saw was sterilized too.

Sizes of extracted bone fragments used for SEM and HRTEM amount to 2.2 mm x 0.1 mm for the longish piece on the left side of Figs. 1a and b and about 1.0 mm x 0.4 mm for the more compact part on the right. The investigation was approved by the local ethical committee of the Justus-Liebig-University Giessen. Small pieces of the trabecular bone were fixed at 4 °C for 24 h in Yellow Fix (4% paraformaldehyde, 2% glutaraldehyde, 0.04% picric acid) and prepared for conventional ultramicrotomy according to a previously published protocol (see Simon *et al.*, 2018)[32]. Staining of semithin sections (1 µm) by means of toluidine blue (Merck, Germany, No 1273) and safranine O (Merck Germany, No. 1382) was performed according to the protocols published by Trump et al.[1], and Ito and Winchester.[2] The investigation was approved by the local ethical committee of the Justus-Liebig-University Giessen.

For sample preparation for rat bone concerning Fig. S5, see citation of Daghma *et al.*[3]

SEM and TEM

SEM investigations were carried out by a Quanta 200 FEGi (FEI, Netherlands) at either 15kV and low vacuum conditions or at 1 kV and high vacuum by using a backscattered electron detector. For electron diffraction, a FEI Tecnai 10 electron microscope (FEI, Eindhoven, Netherlands) with a LaB_6-source at 100 kV acceleration voltage was used. HRTEM was performed by a CM 200 FEG/ Lorentz at 200 kV (FEI, Eindhoven, Netherlands) at 200 kV acceleration voltage with a nominal point resolution of 0.24 nm and line resolution of 0.20 nm. HRTEM data were analyzed by using the Digital Micrograph (Gatan, USA).

Energy Filtered TEM

Energy filtered TEM (EFTEM) images were recorded by a Libra 200 (Carl Zeiss Microscopy GmbH, Oberkochen, Germany) equipped with an omega-type energy filter. The elemental maps were obtained by the 3-window method using 10 eV windows. Care was taken to position the windows correctly (C-K edge and Ca-L edges are close to each other).

Stress modeling

To estimate the stress distribution in lamellar bone with plywood structure caused by a single podosome, we used a simplified cylinder-symmetric model of the podosome structure as shown in Fig. 6a. For determining the stress field in the lamella, the force F_c = 150 nN acting in the central core was given according to reported data. The force F_v in the F-actin cables of the ventral module follows from global force equilibrium. For the sake of simplicity, the lamella was approximated as an isotropic elastic medium, described by an effective Young's modulus E_{eff} and Poisson ratio ν_{eff}. For the calculation, these data were derived from a detailed analysis of the anisotropic elastic constants of lamellar bone with plywood structure.[4] Particularly, we considered the case where the mineralized fibrils are arranged in parallel to the lamella plane, which yields the values E_{eff} = 18 GPa and ν_{eff} = 0.33. Stress fields have been calculated by applying the software Comsol Multiphysics 5.2.

References

(1) Trump, B. F.; Schmuckler, E. A.; Benditt, E. P. A method for staining epoxy sections for light microscopy. *J. Ultrastruct. Res.* **1961**, *5*, 343–348.

(2) Ito, S.; Winchester, R. J. The fine structure of the gastric mucosa in the bat. *J. Cell Biol.* **1963**, *16*, 541–577.

(3) Daghma, D. E. S.; Malhan, D.; Simon P.; Stötzel, S.; Kern, S.; Hassan, F.; Lips, K. S.; · Heiss, C.; Khassawna, T. E. Computational segmentation of collagen fibers in bone matrix indicates bone quality in ovariectomized rat spine. *J. Bone Miner. Metab.* **2018**, *36*, 297–306.

(4) Carnelli D., Vena P., Dao M., Ortiz Ch., Contro R. Orientation and size-dependent mechanical modulation within individual secondary osteons in cortical bone tissue secondary osteons in cortical bone tissue. *J. R. Soc. Interface* **2013**, *10*, 1–12.
